# Supplementary material for: Comprehensive genome-wide association study of different forms of hernia identifies more than 80 associated loci
Source: Nat Commun. 2022 Jun 9;13:3200. doi: 10.1038/s41467-022-30921-4 (PMC9184475; doi:10.1038/s41467-022-30921-4)
Supplement: Supplementary file 1 — Supplementary Information [file 41467_2022_30921_MOESM1_ESM.pdf]

Supplementary Information for

Fadista et al.:

“Comprehensive genome-wide association study of different forms of hernia identifies more than 80 associated loci”

Content pages

Supplementary Figure 1: Quantile-quantile plots for the GWAS scans in SAIGE 2 - 9

Supplementary Figure 2: Miami plots for the sex-specific GWAS scans in SAIGE 10 - 17

Supplementary Figure 3: DEPICT geneset enrichment plots 18 - 20

Supplementary Note 1: Sensitivity analysis for the diverticular disease study 21

Supplementary Note 2: Mendelian randomization of major depression variants on hernia 22 - 23

Supplementary Note 3: Contributors from the FinnGen Consortium 24 - 41

Supplementary Figure 1: Quantile-quantile plots for the GWAS scans in SAIGE

Displayed are expected vs. observed initial (red) and adjusted (blue) $-\log_{10}(P)$, for a) inguinal, b) diaphragmatic, c) umbilical, d) femoral, e) ventral, f) any hernia and g) any hernia excluding diaphragmatic. Observed *P* are truncated at $P = 1 \times 10^{-10}$ for higher resolution of the area where the observed values start to exceed the expected ones. Plots with not truncated *P* are provided in the bottom.

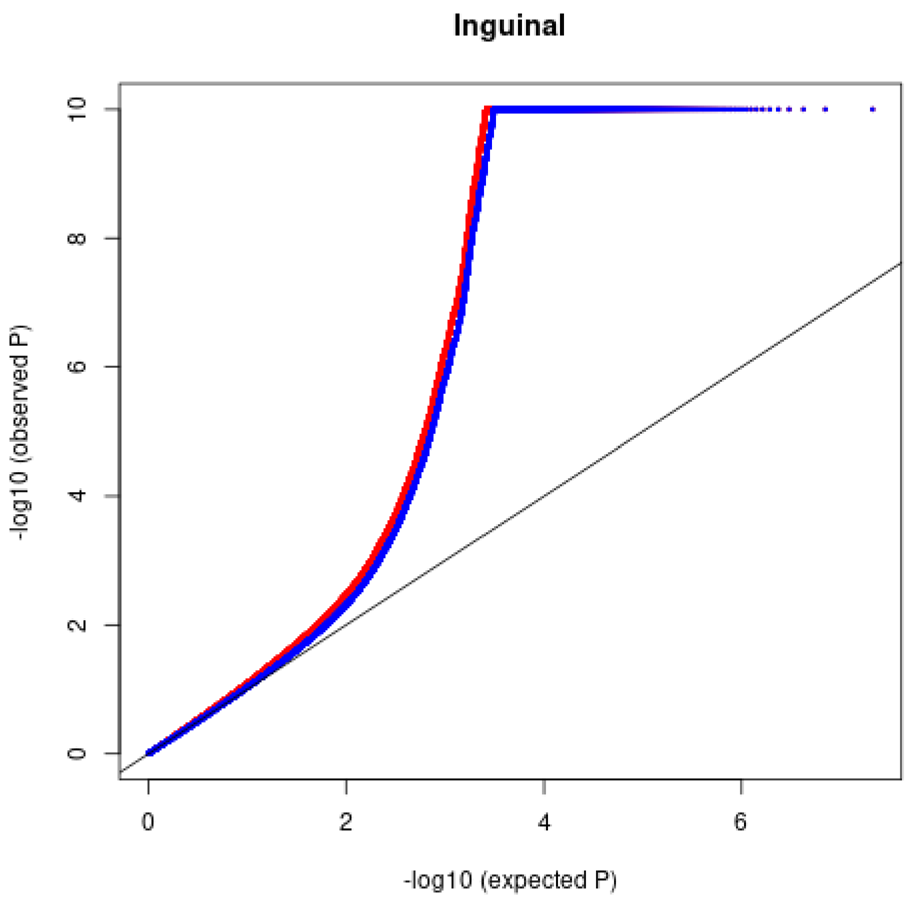

a: genomic control λ = 1.08

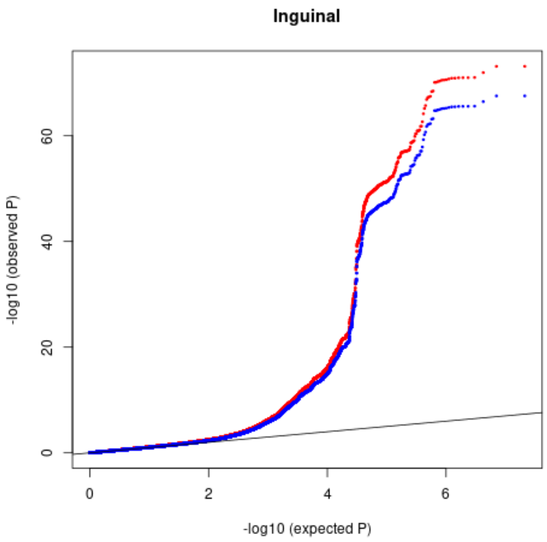

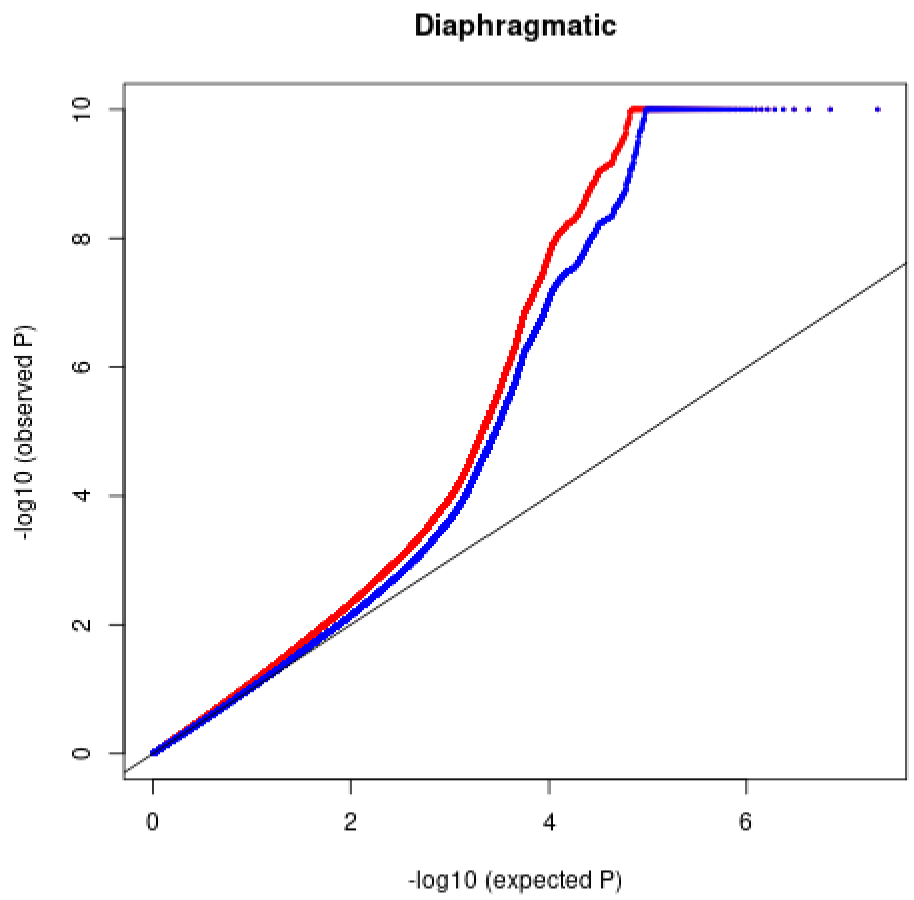

b: genomic control λ = 1.11

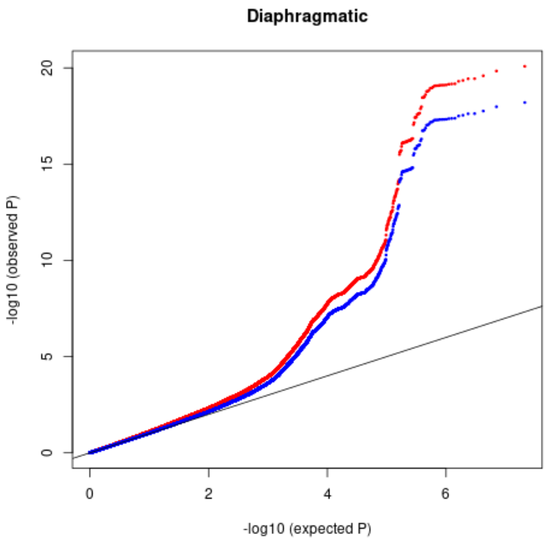

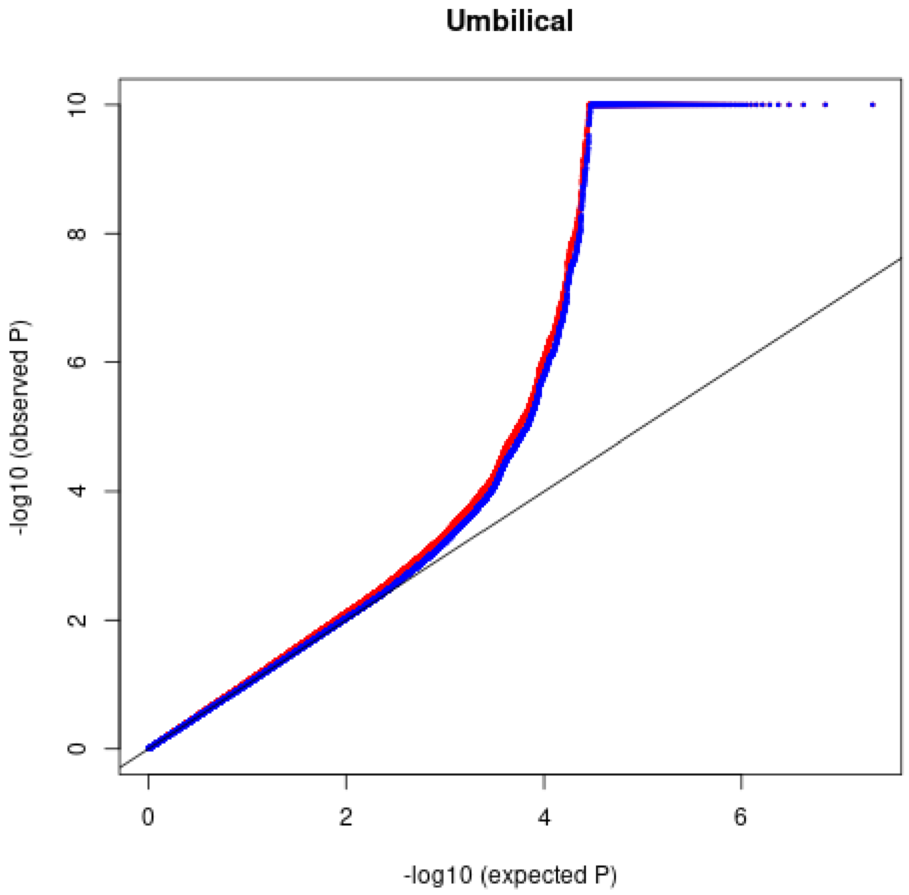

c: genomic control λ = 1.05

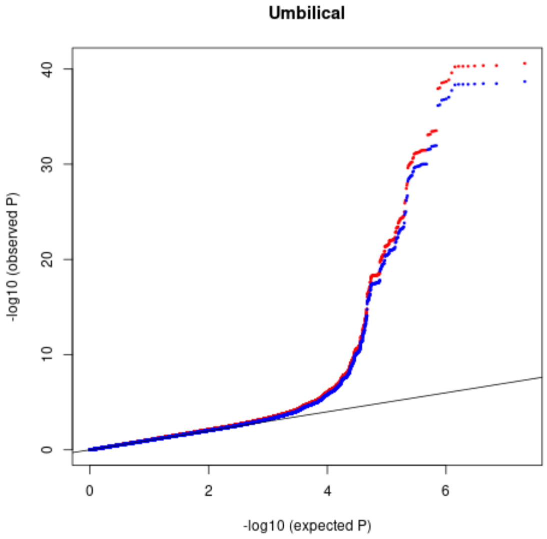

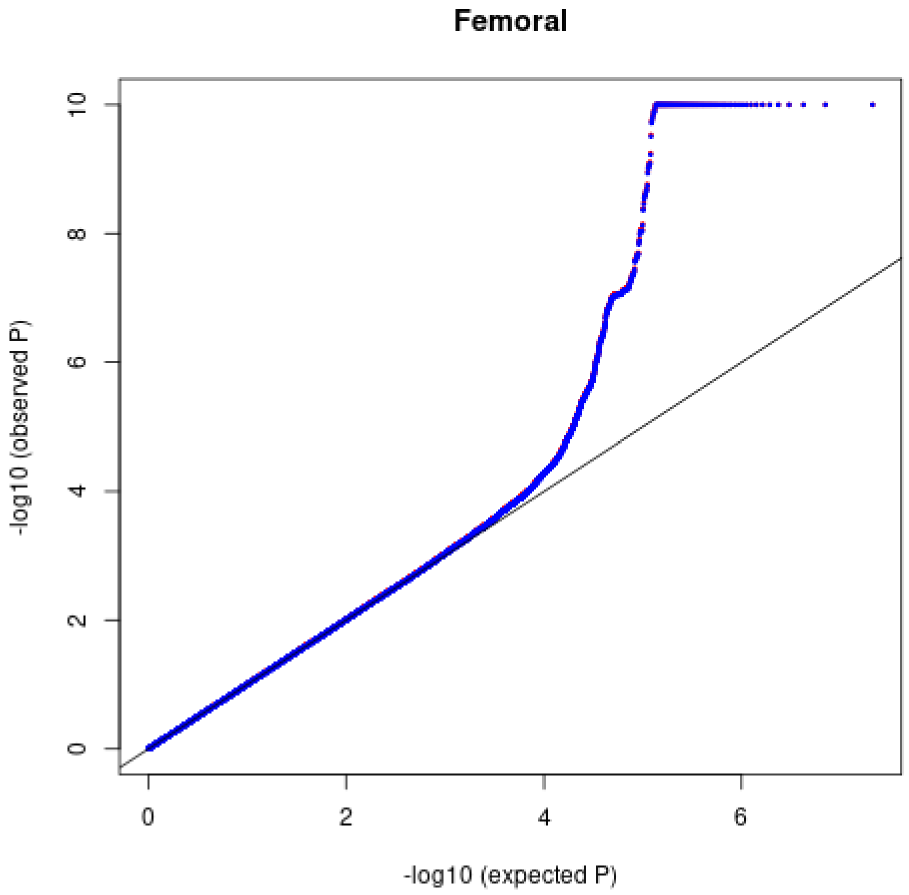

d: genomic control λ = 1.00

NB: λ was 1.003, so the points for the original *P* are hidden behind the ones for the adjusted *P*.

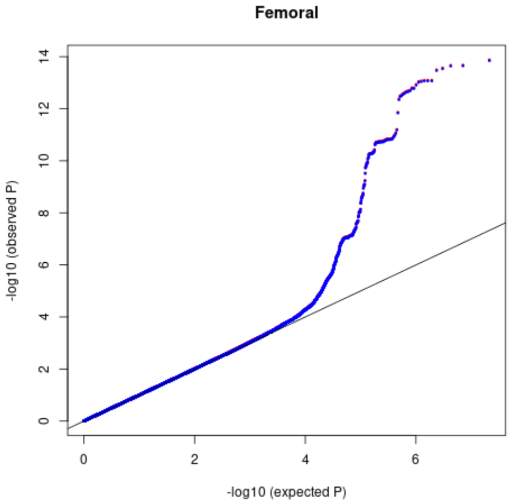

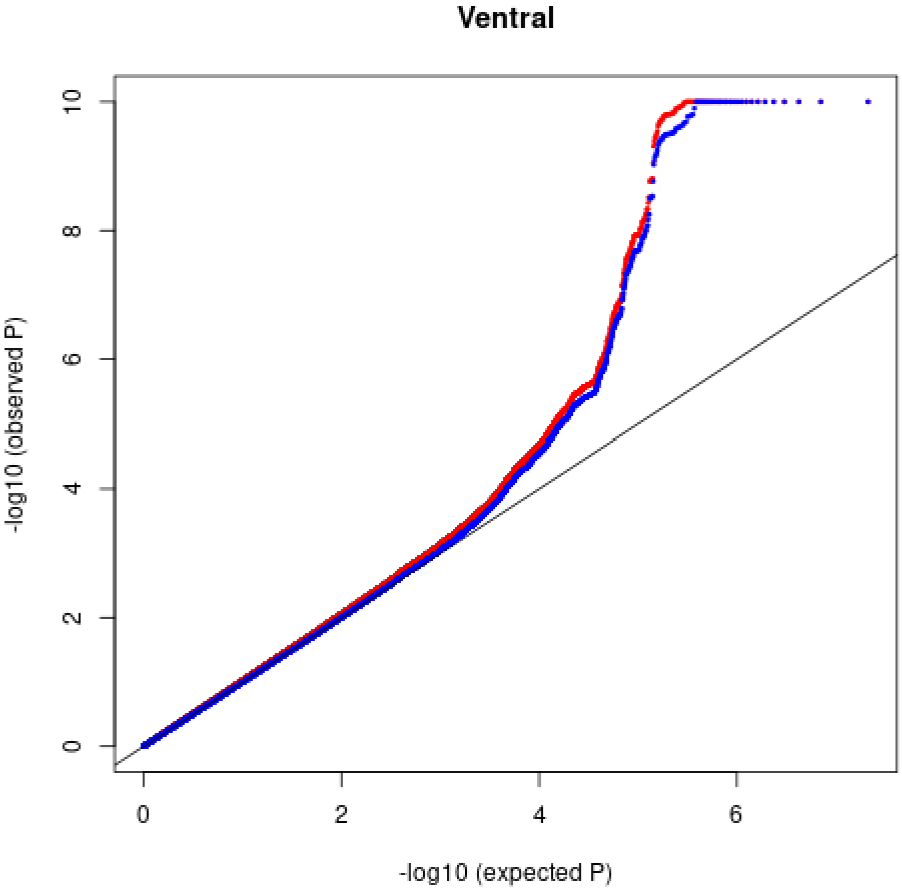

e: genomic control λ = 1.03

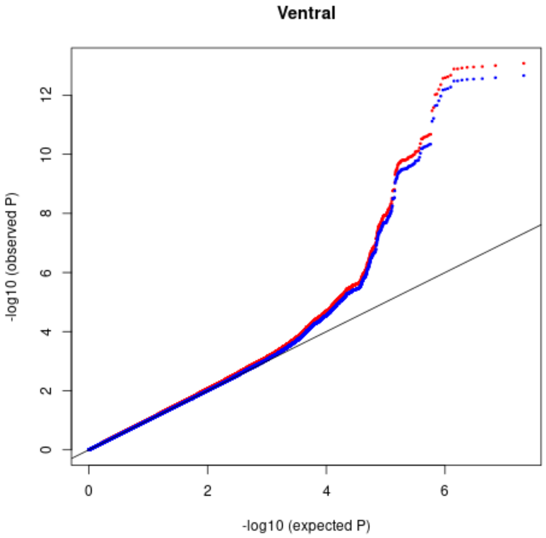

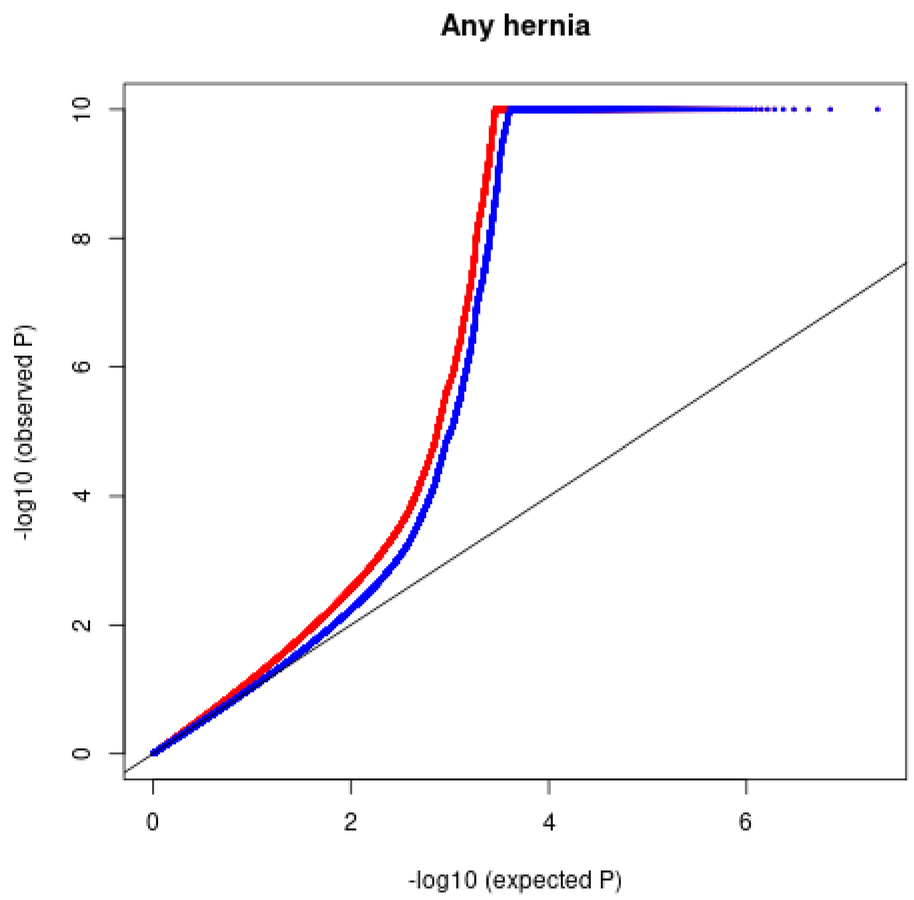

f: genomic control λ = 1.18

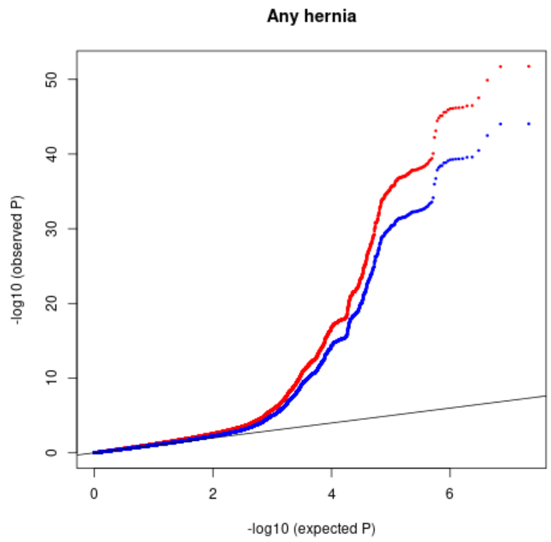

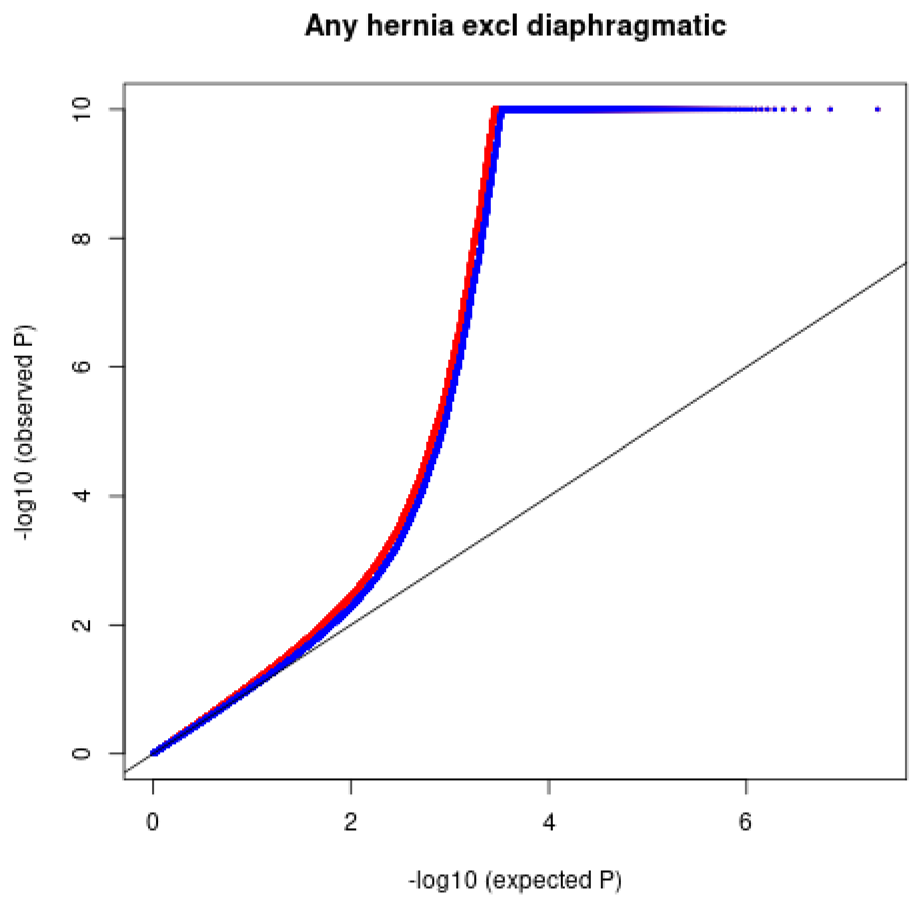

g: genomic control λ = 1.09

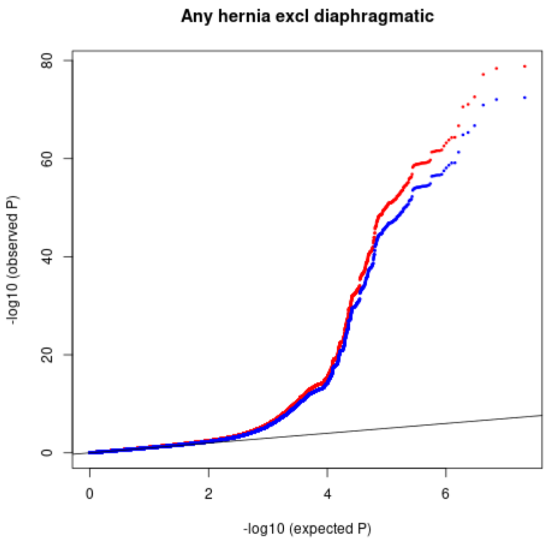

Supplementary Figure 2: Miami plots for the sex-specific GWAS scans in SAIGE

Displayed are adjusted $-\log_{10}(P)$ for males (top) and females (bottom) over the genome for a) inguinal, b) diaphragmatic, c) umbilical, d) femoral, e) ventral, f) any hernia and g) any hernia excluding diaphragmatic.

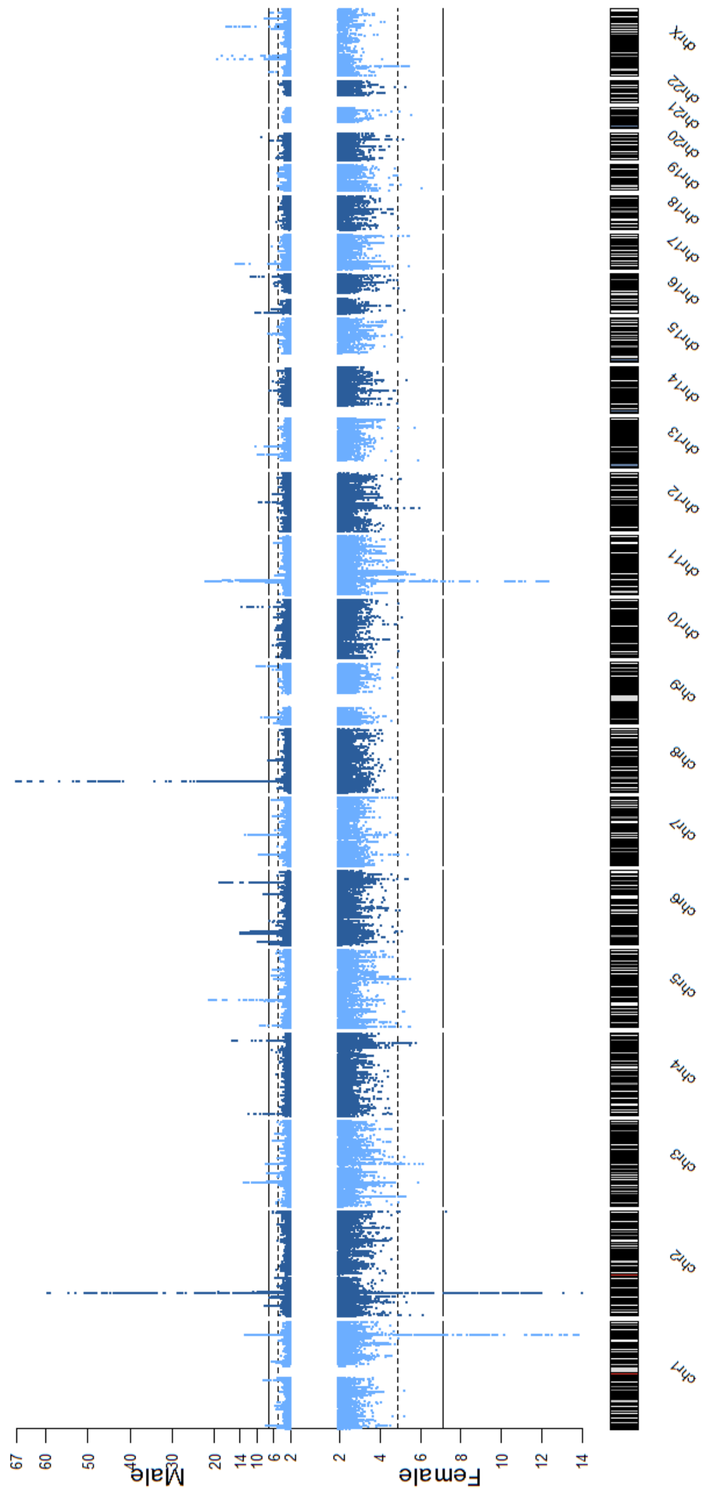

a: Inguinal hernia

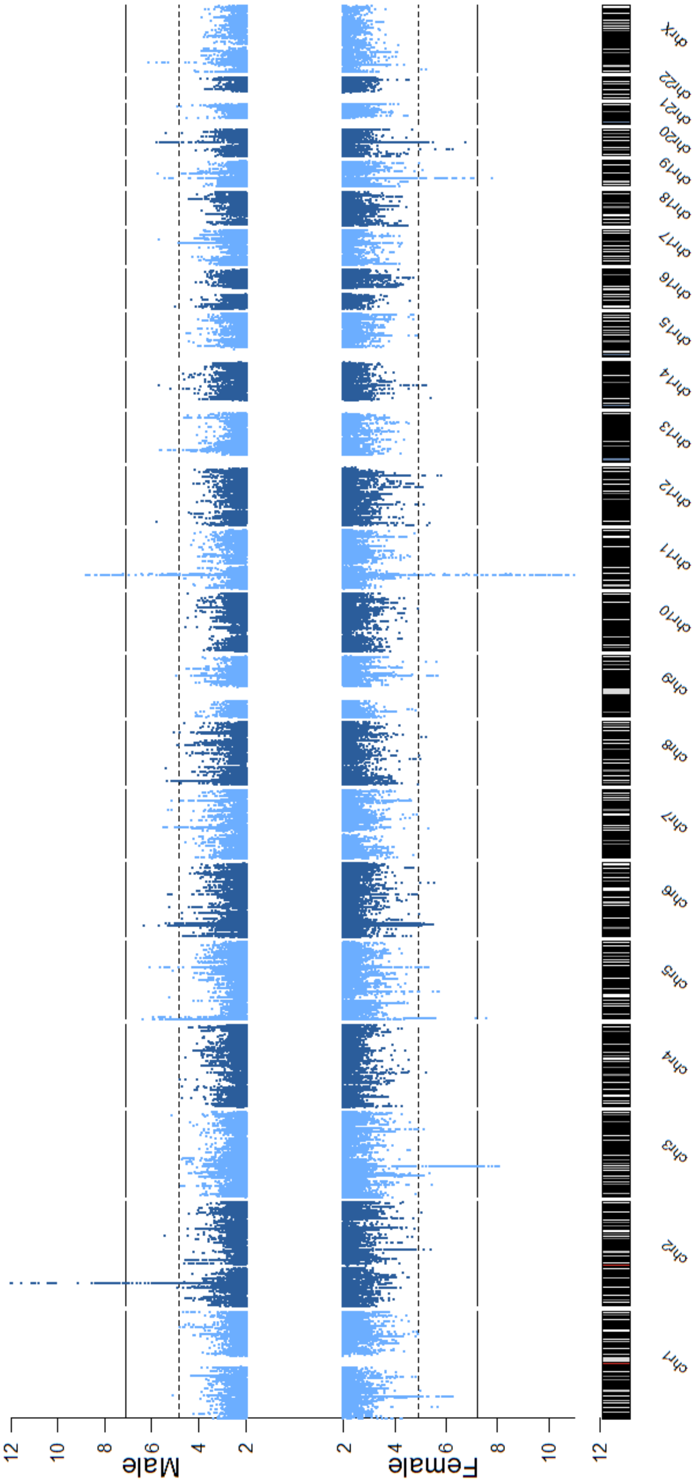

b: Diaphragmatic hernia

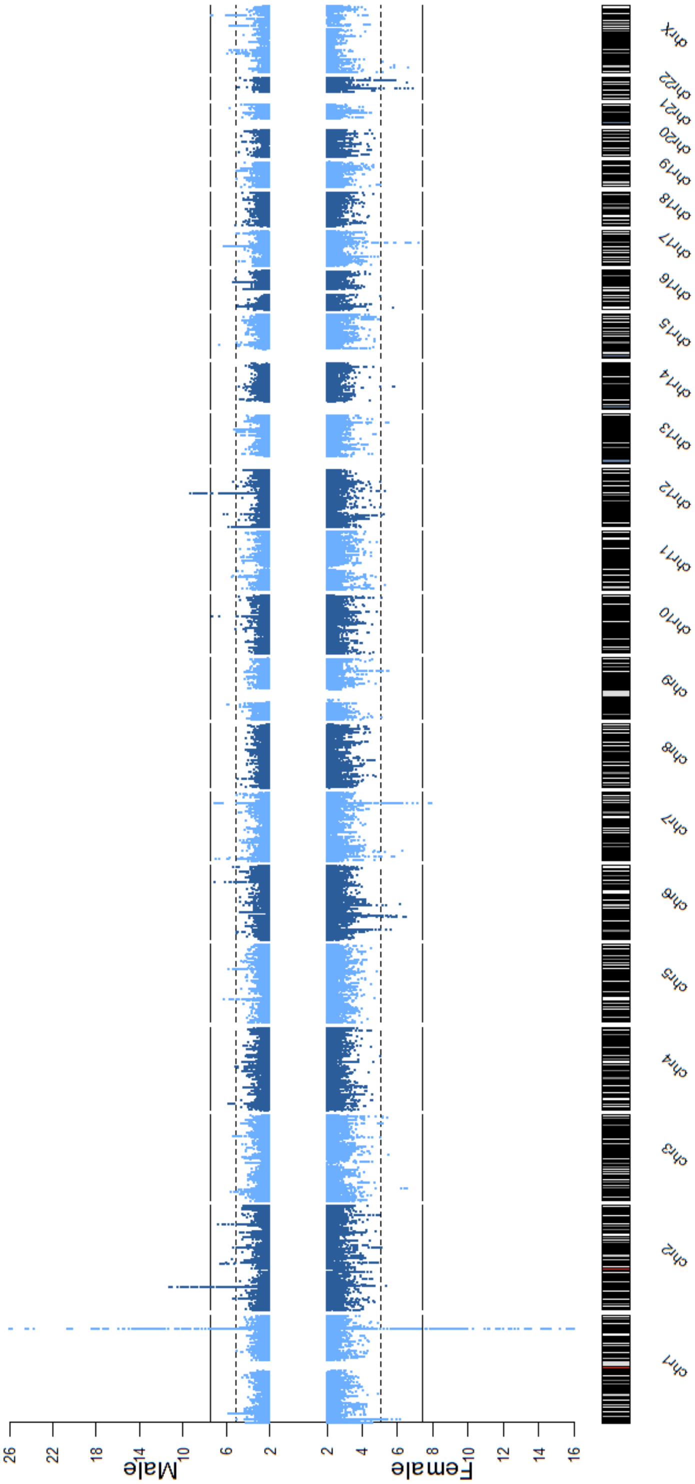

c: Umbilical hernia

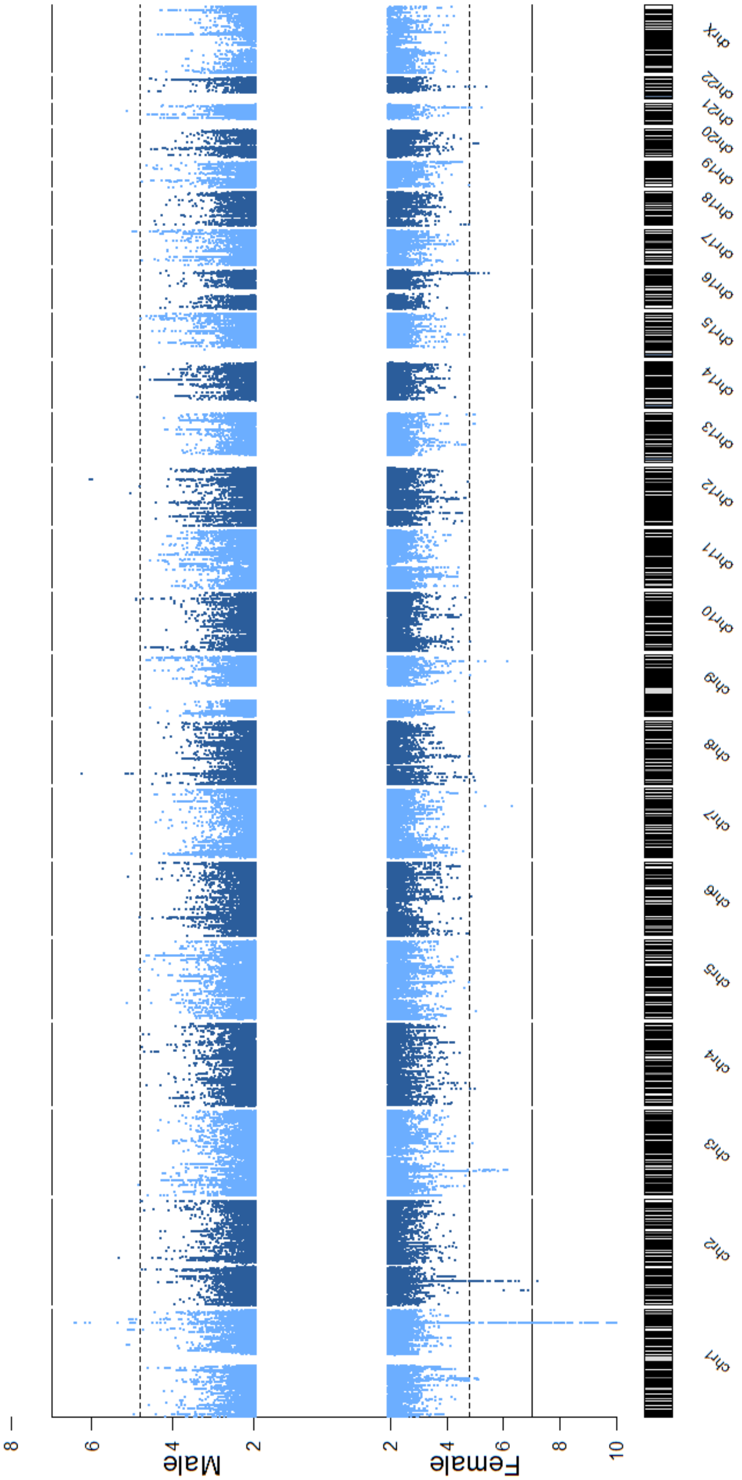

d: Femoral hernia

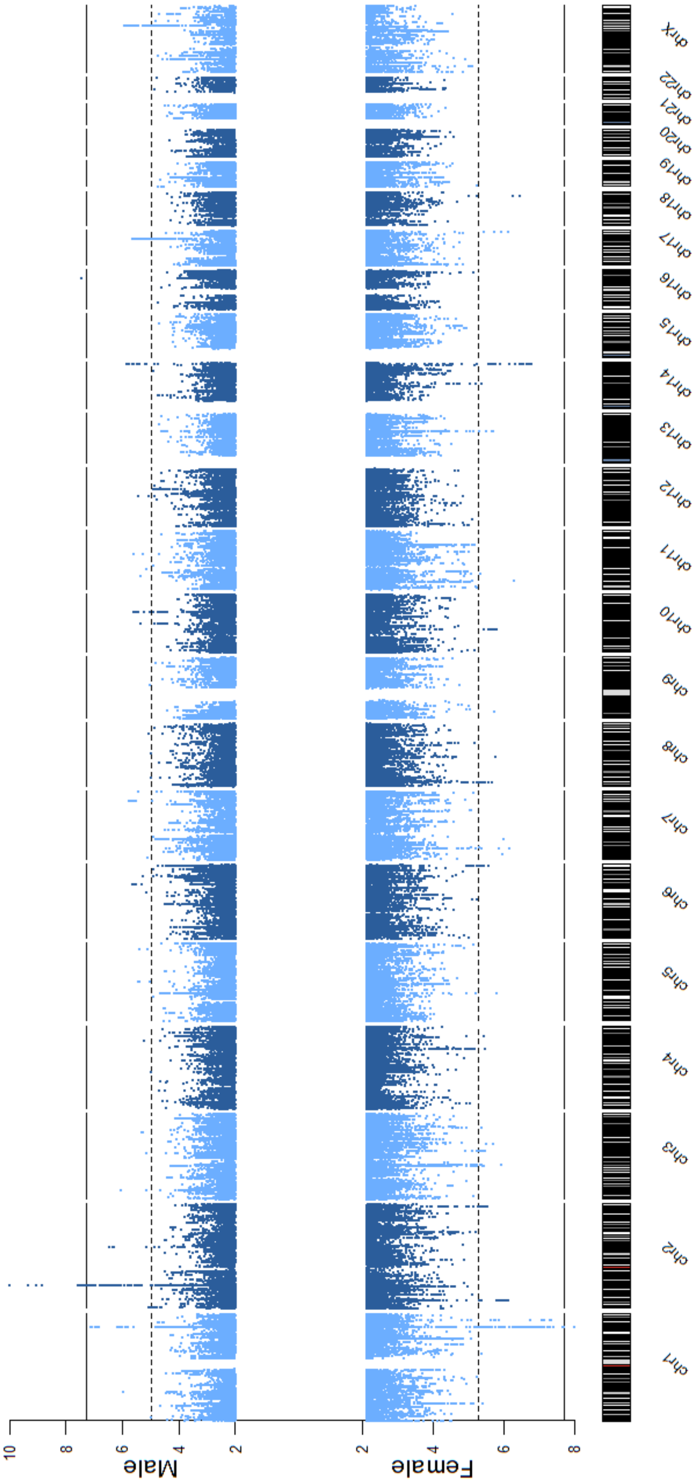

e: Ventral hernia

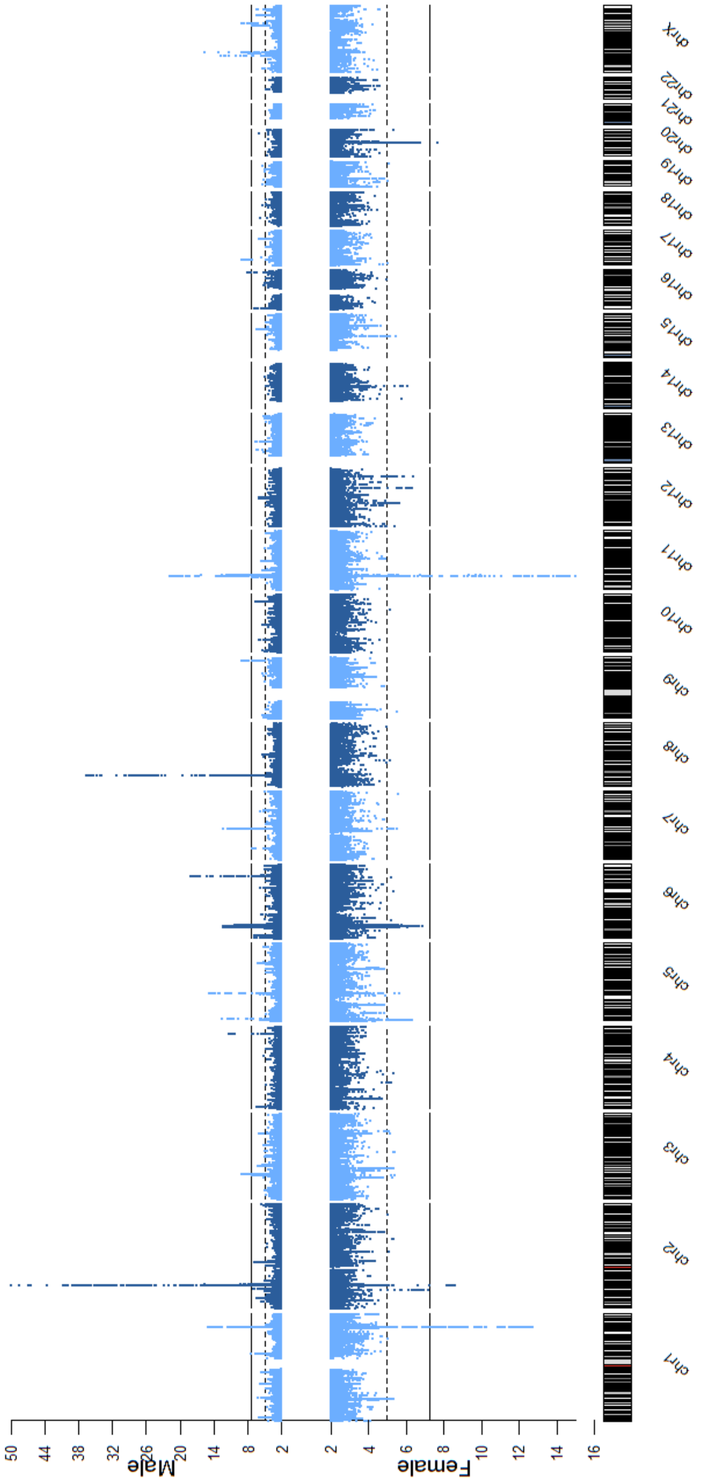
Male
Female
chr1
chr2
chr3
chr4
chr5
chr6
chr7
chr8
chr9
chr10
chr11
chr12
chr13
chr14
chr15
chr16
chr17
chr18
chr19
chr20
chr21
chr22
chrX

f: Any hernia

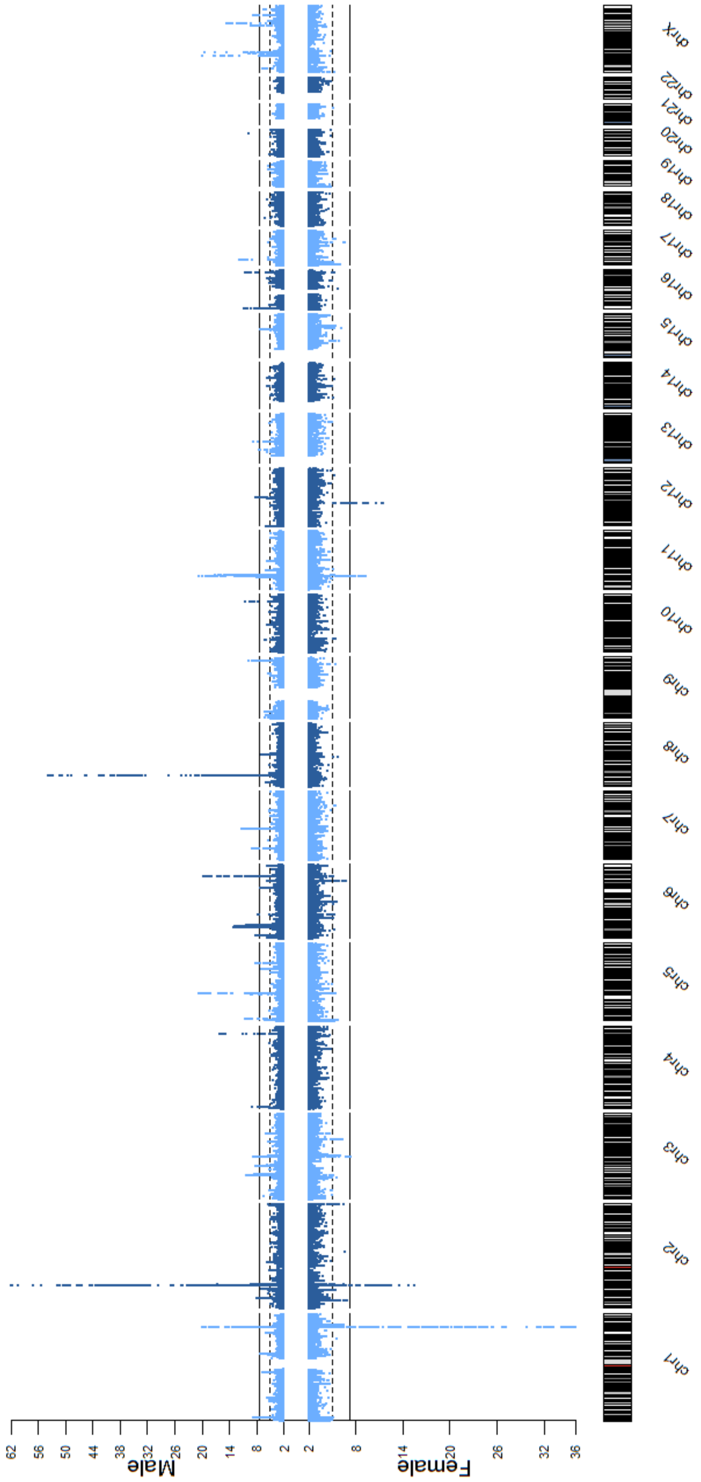

g: Any hernia excluding diaphragmatic

Supplementary Figure 3: DEPICT geneset enrichment plots

Panel a) displays 36 meta genesets derived by clustering the significantly (FDR < 0.05) enriched reconstituted gene sets. The edges connecting the nodes are restricted to r > 0.4.

Panel b) displays 14 gene sets represented by the meta geneset embryonic morphogenesis. derived by clustering the significantly (FDR < 0.05) enriched reconstituted gene sets. The edges connecting the nodes are restricted to r > 0.3.

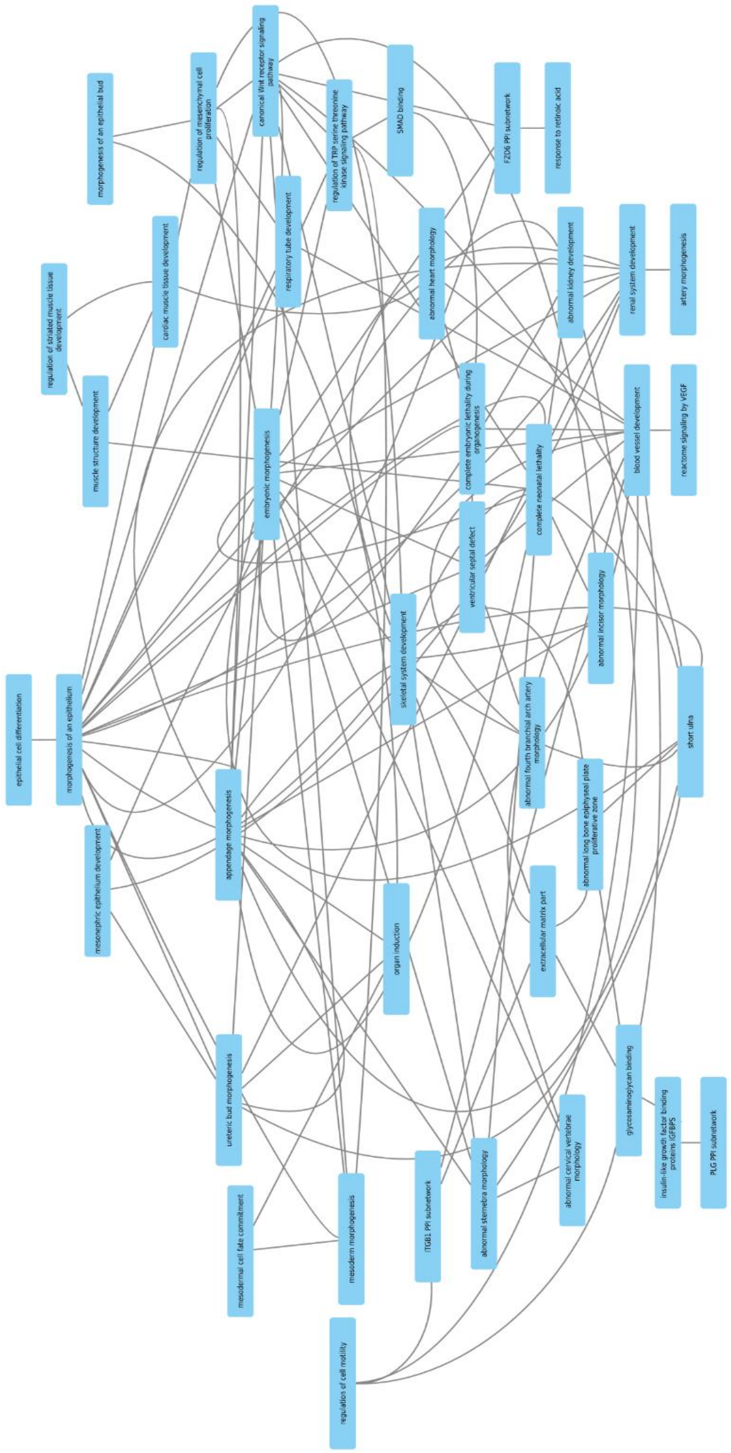

a: DEPICT meta genesets

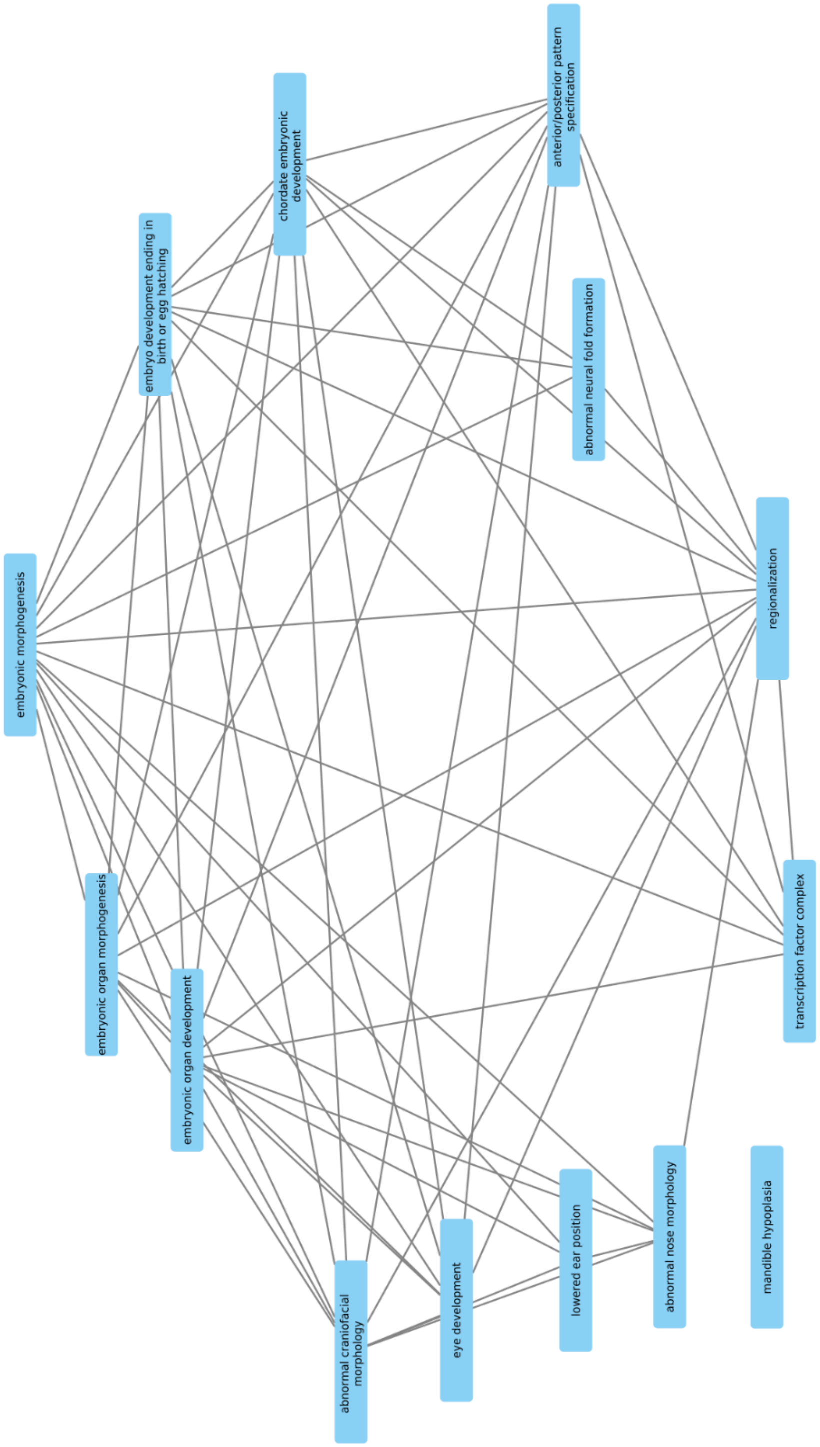

b: DEPICT meta geneset embryonic morphogenesis.

Supplementary Note 1: Sensitivity analysis for the diverticular disease study

One of the follow-up studies was based on a diverticular disease (DD) study. The study was not part of the initial GWAS, so that a bias towards DD loci when selecting the variants for follow-up can be ruled out. We did a sensitivity analysis (**Supplementary Table 3**, sheet "sensitivity for DD") to check whether the inclusion of the DD study biased the results of the follow-up study.

First, we present the results for the DD study separately. Additionally, we did a combined analysis of the follow-up studies without the DD study and a meta-analysis without the DD results, and added the DD study to the results from these analyses to check for heterogeneity that can be directly attributed to the DD study. All analyses were carried out with METAL.

Focusing on the 84 variants with a $P < 5 \times 10^{-8}$ in the meta-analysis of all study groups, we observed five variants with some indication of heterogeneity when adding the DD study to the combined follow-up studies ($I^2 > 70$ and Het P < 0.05, columns AM and AP), for one of these variants (rs250559) the heterogeneity resulted from a substantially larger effect in the DD group. There were four additional variants where the effect in the DD study went the opposite direction, but these effects were not significant (all $P > 0.3$ in the DD analysis). Similar variation in direction of effects was observed for the cohorts from Estonia and iPSYCH (see column X for direction of effects in the three other follow-up cohorts). Overall, 56 variants basically showed no difference between the combined follow-up studies without DD and the DD results ($I^2 = 0$ and Het P > 0.3, again columns AM and AP). The results with regard to the meta-analysis were very similar.

We also investigated whether the results in the DD study biased the results towards the null, i.e. if additional variants reached genome-wide significance in the meta-analysis of the UK Biobank plus the three other studies. Here, rs200219556 with a *P* slightly above 5×10^{-8} in the meta-analysis just reached genome-wide significance in the meta-analysis without DD (column AY), whereas there were two other SNPs with *P* also around the threshold (rs4332926 and rs9455899) that were only genome-wide significant when the DD study was included.

Supplementary Note 2: Mendelian randomization of major depression variants on hernia

We further investigated the link between major depressive disorder (MDD) and hernia by two-sample Mendelian randomization (MR) under the assumption that genetic variants identified in the largest GWAS of MDD directly capture the genetic risk factors for depression. We present results from inverse-variance weighted (IVW), simple median (weighted median with simple median weighting), MR-Egger and MR-PRESSO (see Online Methods for details), with the three latter methods providing important sensitivity analyses. The simple median method gives consistent results even when up to 50% of the information comes from invalid instrumental variables. The MR-Egger intercept test investigates directional pleiotropy based on the significance of the intercept term in the model. In the case of pleiotropy, the genetic variants are not all valid instrumental variables and the standard IVW method estimate is biased. Then, the MR-Egger effect estimate provides a consistent estimate of the causal effect under the InSIDE (Instrument Strength Independent of Direct Effect) assumption. MR-PRESSO includes the global test, where significant findings indicate pleiotropy. The MR-PRESSO outlier test identifies variants with horizontal pleiotropy and excludes them from the instrumental variables in the MR-PRESSO model. Finally, the distortion test tests for a significant change of the effect estimate between IVW and MR-PRESSO due to the removal of outliers (the MR-PRESSO estimate is the same as the IVW one if no outliers are removed). Ideally all four estimates are close to each other and the tests detect no indication of pleiotropy.

The results are displayed in Supplementary Table 7 (Sheet Mendelian Randomization). We found no indication of an effect of MDD on umbilical or ventral hernia (in line with the genetic correlation results). For inguinal hernia, an increased OR was detected with the IVW and MR-Egger methods. However, the MR-PRESSO global and distortion tests indicated pleiotropic effects of some variants distorting the IVW results, and the MR-PRESSO estimate (excluding outliers) was not significant, which was also the case for the simple median estimate. For diaphragmatic hernia, IVW, simple median and MR-PRESSO found highly significant increased ORs, with the MR-PRESSO global test indicating pleiotropy, but there was no distortion. The MR-Egger intercept test also detected pleiotropy and the OR estimate was lower than the ones by the other methods and not significant. Combined the results provide some support for a causal effect of MDD on diaphragmatic hernia.

For ventral hernia, increased ORs were also observed with IVW, simple median and MR-PRESSO, with *P* values ranging from 0.01 to 0.09. Again, the MR-PRESSO tests indicated pleiotropy but no distortion. The MR-Egger intercept test was not significant, and the increased OR was neither. It has to be kept in mind that the ventral hernia GWAS had 4,644 cases, far less than the diaphragmatic hernia GWAS with 31,193. Thus, the power to identify a significant effect of MDD via MR is much lower for this hernia type.

Overall, the MR results provide support that the observed genetic correlations between MDD and diaphragmatic / ventral hernia are at least partly caused by a causal effect of MDD on these forms of hernia. However, as the tests also indicated pleiotropy other factors should be kept in mind. For example, overweight was among the traits showing positive genetic correlation with diaphragmatic and ventral hernia. An MR study of anthropometric traits and depression[1] saw an effect of traits related to overweight on depression but not vice versa. Maybe the interplay between diaphragmatic hernia, depression, overweight and other factors is too complex to be disentangled by MR methods alone, and large longitudinal studies starting in childhood are needed to come to a conclusion here.

Supplementary Reference

1. Speed, M. S., Jefsen, O. H., Børglum, A. D., Speed, D. & Østergaard, S. D. Investigating the association between body fat and depression via Mendelian randomization. *Transl. Psychiatry 2019 91* **9**, 1–9 (2019).

Supplementary Note 3: Contributors from the FinnGen Consortium

Steering Committee

Aarno Palotie	Institute for Molecular Medicine Finland, HiLIFE, University of Helsinki, Finland
Mark Daly	Institute for Molecular Medicine Finland, HiLIFE, University of Helsinki, Finland

Pharmaceutical companies

Bridget Riley-Gills	Abbvie, Chicago, IL, United States
Howard Jacob	Abbvie, Chicago, IL, United States
Dirk Paul	Astra Zeneca, Cambridge, United Kingdom
Heiko Runz	Biogen, Cambridge, MA, United States
Sally John	Biogen, Cambridge, MA, United States
Robert Plenge	Celgene, Summit, NJ, United States/Bristol Myers Squibb, New York, NY, United States
Mark McCarthy	Genentech, San Francisco, CA, United States
Julie Hunkapiller	Genentech, San Francisco, CA, United States
Meg Ehm	GlaxoSmithKline, Brentford, United Kingdom
Kirsi Auro	GlaxoSmithKline, Brentford, United Kingdom
Caroline Fox	Merck, Kenilworth, NJ, United States
Anders Mälarstig	Pfizer, New York, NY, United States
Katherine Klinger	Sanofi, Paris, France
Deepak Raipal	Sanofi, Paris, France
Tim Behrens	Maze Therapeutics, San Francisco, CA, United States
Robert Yang	Janssen Biotech, Beerse, Belgium
Richard Siegel	Novartis, Basel, Switzerland

University of Helsinki & Biobanks

Tomi Mäkelä	HiLIFE, University of Helsinki, Finland, Finland
Jaakko Kaprio	Institute for Molecular Medicine Finland, HiLIFE, Helsinki, Finland, Finland
Petri Virolainen	Auria Biobank / University of Turku / Hospital District of Southwest Finland, Turku, Finland
Antti Hakanen	Auria Biobank / University of Turku / Hospital District of Southwest Finland, Turku, Finland
Terhi Kilpi	THL Biobank / The National Institute of Health and Welfare Helsinki, Finland
Markus Perola	THL Biobank / The National Institute of Health and Welfare Helsinki, Finland
Jukka Partanen	Finnish Red Cross Blood Service / Finnish Hematology Registry and Clinical Biobank, Helsinki, Finland

Anne Pitkäranta Helsinki Biobank / Helsinki University and Hospital District of Helsinki and Uusimaa, Helsinki

Juhani Junttila Northern Finland Biobank Borealis / University of Oulu / Northern Ostrobothnia Hospital District, Oulu, Finland

Raisa Serpi Northern Finland Biobank Borealis / University of Oulu / Northern Ostrobothnia Hospital District, Oulu, Finland

Tarja Laitinen Finnish Clinical Biobank Tampere / University of Tampere / Pirkanmaa Hospital District, Tampere, Finland

Johanna Mäkelä Finnish Clinical Biobank Tampere / University of Tampere / Pirkanmaa Hospital District, Tampere, Finland

Veli-Matti Kosma Biobank of Eastern Finland / University of Eastern Finland / Northern Savo Hospital District, Kuopio, Finland

Urho Kujala Central Finland Biobank / University of Jyväskylä / Central Finland Health Care District, Jyväskylä, Finland

Other Experts/ Non-Voting Members

Outi Tuovila Business Finland, Helsinki, Finland

Raimo Pakkanen Business Finland, Helsinki, Finland

Scientific Committee

Pharmaceutical companies

Jeffrey Waring Abbvie, Chicago, IL, United States

Ali Abbasi Abbvie, Chicago, IL, United States

Mengzhen Liu Abbvie, Chicago, IL, United States

Ioanna Tachmazidou Astra Zeneca, Cambridge, United Kingdom

Chia-Yen Chen Biogen, Cambridge, MA, United States

Heiko Runz Biogen, Cambridge, MA, United States

Shameek Biswas Celgene, Summit, NJ, United States/Bristol Myers Squibb, New York, NY, United States

Julie Hunkapiller Genentech, San Francisco, CA, United States

Meg Ehm GlaxoSmithKline, Brentford, United Kingdom

Neha Raghavan Merck, Kenilworth, NJ, United States

Adriana Huertas-Vazquez Merck, Kenilworth, NJ, United States

Anders Mälarstig Pfizer, New York, NY, United States

Xinli Hu Pfizer, New York, NY, United States

Katherine Klinger Sanofi, Paris, France

Matthias Gossel Sanofi, Paris, France
Robert Graham Maze Therapeutics, San Francisco, CA, United States
Tim Behrens Maze Therapeutics, San Francisco, CA, United States
Beryl Cummings Maze Therapeutics, San Francisco, CA, United States
Wilco Fleuren Janssen Biotech, Beerse, Belgium
Dawn Waterworth Janssen Biotech, Beerse, Belgium
Nicole Renaud Novartis, Basel, Switzerland
Aviv Madar Novartis, Basel, Switzerland
Maen Obeidat Novartis, Basel, Switzerland

University of Helsinki & Biobanks
Samuli Ripatti Institute for Molecular Medicine Finland, HiLIFE, Helsinki, Finland
Johanna Schleutker Auria Biobank / Univ. of Turku / Hospital District of Southwest Finland, Turku, Finland
Markus Perola THL Biobank / The National Institute of Health and Welfare Helsinki, Finland
Mikko Arvas Finnish Red Cross Blood Service / Finnish Hematology Registry and Clinical Biobank, Helsinki, Finland
Olli Carpén Helsinki Biobank / Helsinki University and Hospital District of Helsinki and Uusimaa, Helsinki
Reetta Hinttala Northern Finland Biobank Borealis / University of Oulu / Northern Ostrobothnia Hospital District, Oulu, Finland
Johannes Kettunen Northern Finland Biobank Borealis / University of Oulu / Northern Ostrobothnia Hospital District, Oulu, Finland
Johanna Mäkelä Finnish Clinical Biobank Tampere / University of Tampere / Pirkanmaa Hospital District, Tampere, Finland
Arto Mannermaa Biobank of Eastern Finland / University of Eastern Finland / Northern Savo Hospital District, Kuopio, Finland
Jari Laukkanen Central Finland Biobank / University of Jyväskylä / Central Finland Health Care District, Jyväskylä, Finland
Urho Kujala Central Finland Biobank / University of Jyväskylä / Central Finland Health Care District, Jyväskylä, Finland

Clinical Groups
Neurology Group
Reetta Kälviäinen Northern Savo Hospital District, Kuopio, Finland
Valtteri Julkunen Northern Savo Hospital District, Kuopio, Finland

Hilkka Soininen	Northern Savo Hospital District, Kuopio, Finland
Anne Remes	Northern Ostrobothnia Hospital District, Oulu, Finland
Mikko Hiltunen	Northern Savo Hospital District, Kuopio, Finland
Jukka Peltola	Pirkanmaa Hospital District, Tampere, Finland
Pentti Tienari	Hospital District of Helsinki and Uusimaa, Helsinki, Finland
Juha Rinne	Hospital District of Southwest Finland, Turku, Finland
Roosa Kallionpää	Hospital District of Southwest Finland, Turku, Finland
Ali Abbasi	Abbvie, Chicago, IL, United States
Adam Ziemann	Abbvie, Chicago, IL, United States
Jeffrey Waring	Abbvie, Chicago, IL, United States
Sahar Esmaeeli	Abbvie, Chicago, IL, United States
Nizar Smaoui	Abbvie, Chicago, IL, United States
Anne Lehtonen	Abbvie, Chicago, IL, United States
Susan Eaton	Biogen, Cambridge, MA, United States
Heiko Runz	Biogen, Cambridge, MA, United States
Sanni Lahdenperä	Biogen, Cambridge, MA, United States
Janet van Adelsberg	Celgene, Summit, NJ, United States/ Bristol Myers Squibb, New York, NY, United States
Shameek Biswas	Celgene, Summit, NJ, United States/ Bristol Myers Squibb, New York, NY, United States
Julie Hunkapiller	Genentech, San Francisco, CA, United States
Natalie Bowers	Genentech, San Francisco, CA, United States
Edmond Teng	Genentech, San Francisco, CA, United States
Sarah Pendergrass	Genentech, San Francisco, CA, United States
Onuralp Soylemez	Merck, Kenilworth, NJ, United States
Kari Linden	Pfizer, New York, NY, United States
Fanli Xu	GlaxoSmithKline, Brentford, United Kingdom
David Pulford	GlaxoSmithKline, Brentford, United Kingdom
Kirsi Auro	GlaxoSmithKline, Brentford, United Kingdom
Laura Addis	GlaxoSmithKline, Brentford, United Kingdom
John Eicher	GlaxoSmithKline, Brentford, United Kingdom
Minna Raivio	Hospital District of Helsinki and Uusimaa, Helsinki, Finland
Sarah Pendergrass	Genentech, San Francisco, CA, United States
Beryl Cummings	Maze Therapeutics, San Francisco, CA, United States
Juulia Partanen	Institute for Molecular Medicine Finland, HiLIFE, University of Helsinki, Finland

Gastroenterology Group

Martti Färkkilä	Hospital District of Helsinki and Uusimaa, Helsinki, Finland
Jukka Koskela	Hospital District of Helsinki and Uusimaa, Helsinki, Finland
Sampsa Pikkarainen	Hospital District of Helsinki and Uusimaa, Helsinki, Finland
Airi Jussila	Pirkanmaa Hospital District, Tampere, Finland
Katri Kaukinen	Pirkanmaa Hospital District, Tampere, Finland
Timo Blomster	Northern Ostrobothnia Hospital District, Oulu, Finland
Mikko Kiviniemi	Northern Savo Hospital District, Kuopio, Finland
Markku Voutilainen	Hospital District of Southwest Finland, Turku, Finland
Ali Abbasi	Abbvie, Chicago, IL, United States
Graham Heap	Abbvie, Chicago, IL, United States
Jeffrey Waring	Abbvie, Chicago, IL, United States
Nizar Smaoui	Abbvie, Chicago, IL, United States
Fedik Rahimov	Abbvie, Chicago, IL, United States
Anne Lehtonen	Abbvie, Chicago, IL, United States
Keith Usiskin	Celgene, Summit, NJ, United States/ Bristol Myers Squibb, New York, NY, United States
Tim Lu	Genentech, San Francisco, CA, United States
Natalie Bowers	Genentech, San Francisco, CA, United States
Danny Oh	Genentech, San Francisco, CA, United States
Sarah Pendergrass	Genentech, San Francisco, CA, United States
Kirsi Kalpala	Pfizer, New York, NY, United States
Melissa Miller	Pfizer, New York, NY, United States
Xinli Hu	Pfizer, New York, NY, United States
Linda McCarthy	GlaxoSmithKline, Brentford, United Kingdom
Onuralp Soylemez	Merck, Kenilworth, NJ, United States
Mark Daly	Institute for Molecular Medicine Finland, HiLIFE, University of Helsinki, Finland

Rheumatology Group

Kari Eklund	Hospital District of Helsinki and Uusimaa, Helsinki, Finland
Antti Palomäki	Hospital District of Southwest Finland, Turku, Finland
Pia Isomäki	Pirkanmaa Hospital District, Tampere, Finland
Laura Pirilä	Hospital District of Southwest Finland, Turku, Finland

Oili Kaipiainen-Seppänen	Northern Savo Hospital District, Kuopio, Finland
Johanna Huhtakangas	Northern Ostrobothnia Hospital District, Oulu, Finland
Ali Abbasi	Abbvie, Chicago, IL, United States
Jeffrey Waring	Abbvie, Chicago, IL, United States
Fedik Rahimov	Abbvie, Chicago, IL, United States
Apinya Lertratanakul	Abbvie, Chicago, IL, United States
Nizar Smaoui	Abbvie, Chicago, IL, United States
Anne Lehtonen	Abbvie, Chicago, IL, United States
David Close	Astra Zeneca, Cambridge, United Kingdom
Marla Hochfeld	Celgene, Summit, NJ, United States/ Bristol Myers Squibb, New York, NY, United States
Natalie Bowers	Genentech, San Francisco, CA, United States
Sarah Pendergrass	Genentech, San Francisco, CA, United States
Onuralp Soylemez	Merck, Kenilworth, NJ, United States
Kirsi Kalpala	Pfizer, New York, NY, United States
Nan Bing	Pfizer, New York, NY, United States
Xinli Hu	Pfizer, New York, NY, United States
Jorge Esparza Gordillo	GlaxoSmithKline, Brentford, United Kingdom
Kirsi Auro	GlaxoSmithKline, Brentford, United Kingdom
Dawn Waterworth	Janssen Biotech, Beerse, Belgium
Nina Mars	Institute for Molecular Medicine Finland, HiLIFE, Helsinki, Finland

Pulmonology Group

Tarja Laitinen	Pirkanmaa Hospital District, Tampere, Finland
Margit Pelkonen	Northern Savo Hospital District, Kuopio, Finland
Paula Kauppi	Hospital District of Helsinki and Uusimaa, Helsinki, Finland
Hannu Kankaanranta	Pirkanmaa Hospital District, Tampere, Finland
Terttu Harju	Northern Ostrobothnia Hospital District, Oulu, Finland
Riitta Lahesmaa	Hospital District of Southwest Finland, Turku, Finland
Nizar Smaoui	Abbvie, Chicago, IL, United States
Alex Mackay	Astra Zeneca, Cambridge, United Kingdom
Glenda Lassi	Astra Zeneca, Cambridge, United Kingdom
Susan Eaton	Biogen, Cambridge, MA, United States
Steven Greenberg	Celgene, Summit, NJ, United States/ Bristol Myers Squibb, New York, NY, United States

Hubert Chen	Genentech, San Francisco, CA, United States
Sarah Pendergrass	Genentech, San Francisco, CA, United States
Natalie Bowers	Genentech, San Francisco, CA, United States
Joanna Betts	GlaxoSmithKline, Brentford, United Kingdom
Soumitra Ghosh	GlaxoSmithKline, Brentford, United Kingdom
Kirsi Auro	GlaxoSmithKline, Brentford, United Kingdom
Rajashree Mishra	GlaxoSmithKline, Brentford, United Kingdom
Sina Rüeger	Institute for Molecular Medicine Finland, HiLIFE, University of Helsinki, Finland

Cardiometabolic Diseases Group

Teemu Niiranen	The National Institute of Health and Welfare Helsinki, Finland
Felix Vaura	The National Institute of Health and Welfare Helsinki, Finland
Veikko Salomaa	The National Institute of Health and Welfare Helsinki, Finland
Markus Juonala	Hospital District of Southwest Finland, Turku, Finland
Kaj Metsärinne	Hospital District of Southwest Finland, Turku, Finland
Mika Kähönen	Pirkanmaa Hospital District, Tampere, Finland
Juhani Junttila	Northern Ostrobothnia Hospital District, Oulu, Finland
Markku Laakso	Northern Savo Hospital District, Kuopio, Finland
Jussi Pihlajamäki	Northern Savo Hospital District, Kuopio, Finland
Daniel Gordin	Hospital District of Helsinki and Uusimaa, Helsinki, Finland
Juha Sinisalo	Hospital District of Helsinki and Uusimaa, Helsinki, Finland
Marja-Riitta Taskinen	Hospital District of Helsinki and Uusimaa, Helsinki, Finland
Tiinamaija Tuomi	Hospital District of Helsinki and Uusimaa, Helsinki, Finland
Jari Laukkanen	Central Finland Health Care District, Jyväskylä, Finland
Benjamin Challis	Astra Zeneca, Cambridge, United Kingdom
Dirk Paul	Astra Zeneca, Cambridge, United Kingdom
Julie Hunkapiller	Genentech, San Francisco, CA, United States
Natalie Bowers	Genentech, San Francisco, CA, United States
Sarah Pendergrass	Genentech, San Francisco, CA, United States
Onuralp Soylemez	Merck, Kenilworth, NJ, United States
Jaakko Parkkinen	Pfizer, New York, NY, United States
Melissa Miller	Pfizer, New York, NY, United States
Russell Miller	Pfizer, New York, NY, United States

Audrey Chu	GlaxoSmithKline, Brentford, United Kingdom
Kirsi Auro	GlaxoSmithKline, Brentford, United Kingdom
Keith Usiskin	Celgene, Summit, NJ, United States/ Bristol Myers Squibb, New York, NY, United States
Amanda Elliott	Institute for Molecular Medicine Finland, HiLIFE, University of Helsinki, Finland / Broad Institute, Cambridge, MA, United States
Joel Rämö	Institute for Molecular Medicine Finland, HiLIFE, University of Helsinki, Finland
Samuli Ripatti	Institute for Molecular Medicine Finland, HiLIFE, University of Helsinki, Finland
Mary Pat Reeve	Institute for Molecular Medicine Finland, HiLIFE, University of Helsinki, Finland
Sanni Ruotsalainen	Institute for Molecular Medicine Finland, HiLIFE, University of Helsinki, Finland

Oncology Group

Tuomo Meretoja	Hospital District of Helsinki and Uusimaa, Helsinki, Finland
Heikki Joensuu	Hospital District of Helsinki and Uusimaa, Helsinki, Finland
Olli Carpén	Hospital District of Helsinki and Uusimaa, Helsinki, Finland
Lauri Aaltonen	Hospital District of Helsinki and Uusimaa, Helsinki, Finland
Johanna Mattson	Hospital District of Helsinki and Uusimaa, Helsinki, Finland
Annika Auranen	Pirkanmaa Hospital District , Tampere, Finland
Peeter Karihtala	Northern Ostrobothnia Hospital District, Oulu, Finland
Saila Kauppila	Northern Ostrobothnia Hospital District, Oulu, Finland
Päivi Auvinen	Northern Savo Hospital District, Kuopio, Finland
Klaus Elenius	Hospital District of Southwest Finland, Turku, Finland
Johanna Schleutker	Hospital District of Southwest Finland, Turku, Finland
Relja Popovic	Abbvie, Chicago, IL, United States
Jeffrey Waring	Abbvie, Chicago, IL, United States
Bridget Riley-Gillis	Abbvie, Chicago, IL, United States
Anne Lehtonen	Abbvie, Chicago, IL, United States
Jennifer Schutzman	Genentech, San Francisco, CA, United States
Julie Hunkapiller	Genentech, San Francisco, CA, United States
Natalie Bowers	Genentech, San Francisco, CA, United States
Sarah Pendergrass	Genentech, San Francisco, CA, United States
Andrey Loboda	Merck, Kenilworth, NJ, United States
Aparna Chhibber	Merck, Kenilworth, NJ, United States
Heli Lehtonen	Pfizer, New York, NY, United States
Stefan McDonough	Pfizer, New York, NY, United States

Marika Crohns Sanofi, Paris, France
Sauli Vuoti Sanofi, Paris, France
Diptee Kulkarni GlaxoSmithKline, Brentford, United Kingdom
Kirsi Auro GlaxoSmithKline, Brentford, United Kingdom
Esa Pitkänen Institute for Molecular Medicine Finland, HiLIFE, University of Helsinki, Finland
Nina Mars Institute for Molecular Medicine Finland, HiLIFE, University of Helsinki, Finland
Mark Daly Institute for Molecular Medicine Finland, HiLIFE, University of Helsinki, Finland

Opthalmology Group
Kai Kaarniranta Northern Savo Hospital District, Kuopio, Finland
Joni A Turunen Hospital District of Helsinki and Uusimaa, Helsinki, Finland
Terhi Ollila Hospital District of Helsinki and Uusimaa, Helsinki, Finland
Sanna Seitsonen Hospital District of Helsinki and Uusimaa, Helsinki, Finland
Hannu Uusitalo Pirkanmaa Hospital District, Tampere, Finland
Vesa Aaltonen Hospital District of Southwest Finland, Turku, Finland
Hannele Uusitalo-Järvinen Pirkanmaa Hospital District, Tampere, Finland
Marja Luodonpää Northern Ostrobothnia Hospital District, Oulu, Finland
Nina Hautala Northern Ostrobothnia Hospital District, Oulu, Finland
Mengzhen Liu Abbvie, Chicago, IL, United States
Heiko Runz Biogen, Cambridge, MA, United States
Stephanie Loomis Biogen, Cambridge, MA, United States
Erich Strauss Genentech, San Francisco, CA, United States
Natalie Bowers Genentech, San Francisco, CA, United States
Hao Chen Genentech, San Francisco, CA, United States
Sarah Pendergrass Genentech, San Francisco, CA, United States
Anna Podgornaia Merck, Kenilworth, NJ, United States
Juha Karjalainen Institute for Molecular Medicine Finland, HiLIFE, University of Helsinki, Finland / Broad Institute, Cambridge, MA, United States
Esa Pitkänen Institute for Molecular Medicine Finland, HiLIFE, University of Helsinki, Finland

Dermatology Group
Kaisa Tasanen Northern Ostrobothnia Hospital District, Oulu, Finland
Laura Huilaja Northern Ostrobothnia Hospital District, Oulu, Finland
Katariina Hannula-Jouppi Hospital District of Helsinki and Uusimaa, Helsinki, Finland

Teea Salmi	Pirkanmaa Hospital District, Tampere, Finland
Sirkku Peltonen	Hospital District of Southwest Finland, Turku, Finland
Leena Koulu	Hospital District of Southwest Finland, Turku, Finland
Kirsi Kalpala	Pfizer, New York, NY, United States
Ying Wu	Pfizer, New York, NY, United States
David Choy	Genentech, San Francisco, CA, United States
Sarah Pendergrass	Genentech, San Francisco, CA, United States
Nizar Smaoui	Abbvie, Chicago, IL, United States
Fedik Rahimov	Abbvie, Chicago, IL, United States
Anne Lehtonen	Abbvie, Chicago, IL, United States
Dawn Waterworth	Janssen Biotech, Beerse, Belgium

Odontology Group

Pirkko Pussinen	Hospital District of Helsinki and Uusimaa, Helsinki, Finland
Aino Salminen	Hospital District of Helsinki and Uusimaa, Helsinki, Finland
Tuula Salo	Hospital District of Helsinki and Uusimaa, Helsinki, Finland
David Rice	Hospital District of Helsinki and Uusimaa, Helsinki, Finland
Pekka Nieminen	Hospital District of Helsinki and Uusimaa, Helsinki, Finland
Ulla Palotie	Hospital District of Helsinki and Uusimaa, Helsinki, Finland
Juha Sinisalo	Hospital District of Helsinki and Uusimaa, Helsinki, Finland
Maria Siponen	Northern Savo Hospital District, Kuopio, Finland
Liisa Suominen	Northern Savo Hospital District, Kuopio, Finland
Päivi Mäntylä	Northern Savo Hospital District, Kuopio, Finland
Ulvi Gursoy	Hospital District of Southwest Finland, Turku, Finland
Vuokko Anttonen	Northern Ostrobothnia Hospital District, Oulu, Finland
Kirsi Sipilä	Northern Ostrobothnia Hospital District, Oulu, Finland
Sarah Pendergrass	Genentech, San Francisco, CA, United States

Women's Health and Reproduction Group

Hannele Laivuori	Institute for Molecular Medicine Finland, HiLIFE, University of Helsinki, Finland
Venla Kurra	Pirkanmaa Hospital District, Tampere, Finland
Oskari Heikinheimo	Hospital District of Helsinki and Uusimaa, Helsinki, Finland
Ilkka Kalliala	Hospital District of Helsinki and Uusimaa, Helsinki, Finland

Laura Kotaniemi-Talonen	Pirkanmaa Hospital District, Tampere, Finland
Kari Nieminen	Pirkanmaa Hospital District, Tampere, Finland
Päivi Polo	Hospital District of Southwest Finland, Turku, Finland
Kaarin Mäkikallio	Hospital District of Southwest Finland, Turku, Finland
Eeva Ekholm	Hospital District of Southwest Finland, Turku, Finland
Marja Vääräsmäki	Northern Ostrobothnia Hospital District, Oulu, Finland
Outi Uimari	Northern Ostrobothnia Hospital District, Oulu, Finland
Laure Morin-Papunen	Northern Ostrobothnia Hospital District, Oulu, Finland
Marjo Tuppurainen	Northern Savo Hospital District, Kuopio, Finland
Katja Kivinen	Institute for Molecular Medicine Finland, HiLIFE, University of Helsinki, Finland
Elisabeth Widen	Institute for Molecular Medicine Finland, HiLIFE, University of Helsinki, Finland
Taru Tukiainen	Institute for Molecular Medicine Finland, HiLIFE, University of Helsinki, Finland
Mary Pat Reeve	Institute for Molecular Medicine Finland, HiLIFE, University of Helsinki, Finland
Mark Daly	Institute for Molecular Medicine Finland, HiLIFE, University of Helsinki, Finland
Liu Aoxing	Institute for Molecular Medicine Finland, HiLIFE, University of Helsinki, Finland
Eija Laakkonen	University of Jyväskylä, Jyväskylä, Finland
Niko Välimäki	University of Helsinki, Helsinki, Finland
Lauri Aaltonen	Hospital District of Helsinki and Uusimaa, Helsinki, Finland
Johannes Kettunen	Northern Ostrobothnia Hospital District, Oulu, Finland
Mikko Arvas	Finnish Red Cross Blood Service, Helsinki, Finland
Jeffrey Waring	Abbvie, Chicago, IL, United States
Bridget Riley-Gillis	Abbvie, Chicago, IL, United States
Mengzhen Liu	Abbvie, Chicago, IL, United States
Janet Kumar	GlaxoSmithKline, Brentford, United Kingdom
Kirsi Auro	GlaxoSmithKline, Brentford, United Kingdom
Andrea Ganna	Institute for Molecular Medicine Finland, HiLIFE, University of Helsinki, Finland
Sarah Pendergrass	Genentech, San Francisco, CA, United States

FinnGen Analysis working group

Justin Wade Davis	Abbvie, Chicago, IL, United States
Bridget Riley-Gillis	Abbvie, Chicago, IL, United States
Danjuma Quarless	Abbvie, Chicago, IL, United States
Fedik Rahimov	Abbvie, Chicago, IL, United States

Sahar Esmaeeli	Abbvie, Chicago, IL, United States
Slavé Petrovski	Astra Zeneca, Cambridge, United Kingdom
Eleonor Wigmore	Astra Zeneca, Cambridge, United Kingdom
Adele Mitchell	Biogen, Cambridge, MA, United States
Benjamin Sun	Biogen, Cambridge, MA, United States
Ellen Tsai	Biogen, Cambridge, MA, United States
Denis Baird	Biogen, Cambridge, MA, United States
Paola Bronson	Biogen, Cambridge, MA, United States
Ruoyu Tian	Biogen, Cambridge, MA, United States
Stephanie Loomis	Biogen, Cambridge, MA, United States
Yunfeng Huang	Biogen, Cambridge, MA, United States
Joseph Maranville	Celgene, Summit, NJ, United States/ Bristol Myers Squibb, New York, NY, United States
Shameek Biswas	Celgene, Summit, NJ, United States/ Bristol Myers Squibb, New York, NY, United States
Elmutaz Mohammed	Celgene, Summit, NJ, United States/ Bristol Myers Squibb, New York, NY, United States
Samir Wadhawan	Celgene, Summit, NJ, United States/ Bristol Myers Squibb, New York, NY, United States
Erika Kvikstad	Celgene, Summit, NJ, United States/ Bristol Myers Squibb, New York, NY, United States
Minal Caliskan	Celgene, Summit, NJ, United States/ Bristol Myers Squibb, New York, NY, United States
Diana Chang	Genentech, San Francisco, CA, United States
Julie Hunkapiller	Genentech, San Francisco, CA, United States
Tushar Bhangale	Genentech, San Francisco, CA, United States
Natalie Bowers	Genentech, San Francisco, CA, United States
Sarah Pendergrass	Genentech, San Francisco, CA, United States
Kirill Shkura	Merck, Kenilworth, NJ, United States
Victor Neduva	Merck, Kenilworth, NJ, United States
Xing Chen	Pfizer, New York, NY, United States
Åsa Hedman	Pfizer, New York, NY, United States
Karen S King	GlaxoSmithKline, Brentford, United Kingdom
Padhraig Gormley	GlaxoSmithKline, Brentford, United Kingdom
Jimmy Liu	GlaxoSmithKline, Brentford, United Kingdom
Clarence Wang	Sanofi, Paris, France
Ethan Xu	Sanofi, Paris, France
Franck Auge	Sanofi, Paris, France
Clement Chatelain	Sanofi, Paris, France

Deepak Rajpal Sanofi, Paris, France

Dongyu Liu Sanofi, Paris, France

Katherine Call Sanofi, Paris, France

Tai-He Xia Sanofi, Paris, France

Beryl Cummings Maze Therapeutics, San Francisco, CA, United States

Matt Brauer Maze Therapeutics, San Francisco, CA, United States

Huilei Xu Novartis, Basel, Switzerland

Amy Cole Novartis, Basel, Switzerland

Jonathan Chung Novartis, Basel, Switzerland

Jaison Jacob Novartis, Basel, Switzerland

Katrina de Lange Novartis, Basel, Switzerland

Jonas Zierer Novartis, Basel, Switzerland

Mitja Kurki Institute for Molecular Medicine Finland, HiLIFE, University of Helsinki, Finland / Broad Institute, Cambridge, MA, United States

Samuli Ripatti Institute for Molecular Medicine Finland, HiLIFE, University of Helsinki, Finland

Mark Daly Institute for Molecular Medicine Finland, HiLIFE, University of Helsinki, Finland

Juha Karjalainen Institute for Molecular Medicine Finland, HiLIFE, University of Helsinki, Finland / Broad Institute, Cambridge, MA, United States

Aki Havulinna Institute for Molecular Medicine Finland, HiLIFE, University of Helsinki, Finland

Juha Mehtonen Institute for Molecular Medicine Finland, HiLIFE, University of Helsinki, Finland

Priit Palta Institute for Molecular Medicine Finland, HiLIFE, University of Helsinki, Finland

Shabbeer Hassan Institute for Molecular Medicine Finland, HiLIFE, University of Helsinki, Finland

Pietro Della Briotta Parolo Institute for Molecular Medicine Finland, HiLIFE, University of Helsinki, Finland

Wei Zhou Broad Institute, Cambridge, MA, United States

Mutaamba Maasha Broad Institute, Cambridge, MA, United States

Shabbeer Hassan Institute for Molecular Medicine Finland, HiLIFE, University of Helsinki, Finland

Susanna Lemmelä Institute for Molecular Medicine Finland, HiLIFE, University of Helsinki, Finland

Manuel Rivas University of Stanford, Stanford, CA, United States

Aarno Palotie Institute for Molecular Medicine Finland, HiLIFE, University of Helsinki, Finland

Arto Lehisto Institute for Molecular Medicine Finland, HiLIFE, University of Helsinki, Finland

Andrea Ganna Institute for Molecular Medicine Finland, HiLIFE, University of Helsinki, Finland

Vincent Llorens Institute for Molecular Medicine Finland, HiLIFE, University of Helsinki, Finland

Hannele Laivuori Institute for Molecular Medicine Finland, HiLIFE, University of Helsinki, Finland

Mari E Niemi Institute for Molecular Medicine Finland, HiLIFE, University of Helsinki, Finland

Taru Tukiainen Institute for Molecular Medicine Finland, HiLIFE, University of Helsinki, Finland
Mary Pat Reeve Institute for Molecular Medicine Finland, HiLIFE, University of Helsinki, Finland
Henrike Heyne Institute for Molecular Medicine Finland, HiLIFE, University of Helsinki, Finland
Nina Mars Institute for Molecular Medicine Finland, HiLIFE, University of Helsinki, Finland
Kimmo Palin University of Helsinki, Helsinki, Finland
Javier Garcia-Tabuenca University of Tampere, Tampere, Finland
Harri Siirtola University of Tampere, Tampere, Finland
Tuomo Kiiskinen Institute for Molecular Medicine Finland, HiLIFE, University of Helsinki, Finland
Jiwoo Lee Institute for Molecular Medicine Finland, HiLIFE, University of Helsinki, Finland / Broad Institute, Cambridge, MA, United States
Kristin Tsuo Institute for Molecular Medicine Finland, HiLIFE, University of Helsinki, Finland / Broad Institute, Cambridge, MA, United States
Amanda Elliott Institute for Molecular Medicine Finland, HiLIFE, University of Helsinki, Finland / Broad Institute, Cambridge, MA, United States
Kati Kristiansson THL Biobank / The National Institute of Health and Welfare Helsinki, Finland
Mikko Arvas Finnish Red Cross Blood Service / Finnish Hematology Registry and Clinical Biobank, Helsinki, Finland
Kati Hyvärinen Finnish Red Cross Blood Service, Helsinki, Finland
Jarmo Ritari Finnish Red Cross Blood Service, Helsinki, Finland
Miika Koskinen Helsinki Biobank / Helsinki University and Hospital District of Helsinki and Uusimaa, Helsinki
Olli Carpén Helsinki Biobank / Helsinki University and Hospital District of Helsinki and Uusimaa, Helsinki
Johannes Kettunen Northern Finland Biobank Borealis / University of Oulu / Northern Ostrobothnia Hospital District, Oulu, Finland
Katri Pylkäs University of Oulu, Oulu, Finland
Marita Kalaoja University of Oulu, Oulu, Finland
Minna Karjalainen University of Oulu, Oulu, Finland
Tuomo Mantere Northern Finland Biobank Borealis / University of Oulu / Northern Ostrobothnia Hospital District, Oulu, Finland
Eeva Kangasniemi Finnish Clinical Biobank Tampere / University of Tampere / Pirkanmaa Hospital District, Tampere, Finland
Sami Heikkinen University of Eastern Finland, Kuopio, Finland
Arto Mannermaa Biobank of Eastern Finland / University of Eastern Finland / Northern Savo Hospital District, Kuopio, Finland
Eija Laakkonen University of Jyväskylä, Jyväskylä, Finland

Samuel Heron University of Turku, Turku, Finland
Dhanaprakash Jambulingam University of Turku, Turku, Finland
Venkat Subramaniam Rathinakannan University of Turku, Turku, Finland
Nina Pitkänen Auria Biobank / University of Turku / Hospital District of Southwest Finland, Turku, Finland

Biobank directors
Lila Kallio Auria Biobank / University of Turku / Hospital District of Southwest Finland, Turku, Finland
Sirpa Soini THL Biobank / The National Institute of Health and Welfare Helsinki, Finland
Jukka Partanen Finnish Red Cross Blood Service / Finnish Hematology Registry and Clinical Biobank, Helsinki, Finland
Eero Punkka Helsinki Biobank / Helsinki University and Hospital District of Helsinki and Uusimaa, Helsinki
Raisa Serpi Northern Finland Biobank Borealis / University of Oulu / Northern Ostrobothnia Hospital District, Oulu, Finland
Johanna Mäkelä Finnish Clinical Biobank Tampere / University of Tampere / Pirkanmaa Hospital District, Tampere, Finland
Veli-Matti Kosma Biobank of Eastern Finland / University of Eastern Finland / Northern Savo Hospital District, Kuopio, Finland
Teijo Kuopio Central Finland Biobank / University of Jyväskylä / Central Finland Health Care District, Jyväskylä, Finland

FinnGen Teams
Administration
Anu Jalanko Institute for Molecular Medicine Finland, HiLIFE, University of Helsinki, Finland
Huei-Yi Shen Institute for Molecular Medicine Finland, HiLIFE, University of Helsinki, Finland
Risto Kajanne Institute for Molecular Medicine Finland, HiLIFE, University of Helsinki, Finland
Mervi Aavikko Institute for Molecular Medicine Finland, HiLIFE, University of Helsinki, Finland

Analysis
Mitja Kurki Institute for Molecular Medicine Finland, HiLIFE, University of Helsinki, Finland / Broad Institute, Cambridge, MA, United States
Juha Karjalainen Institute for Molecular Medicine Finland, HiLIFE, University of Helsinki, Finland / Broad Institute, Cambridge, MA, United States

Pietro Della Briotta Parolo Institute for Molecular Medicine Finland, HiLIFE, University of Helsinki, Finland
Arto Lehisto Institute for Molecular Medicine Finland, HiLIFE, University of Helsinki, Finland
Juha Mehtonen Institute for Molecular Medicine Finland, HiLIFE, University of Helsinki, Finland
Wei Zhou Broad Institute, Cambridge, MA, United States
Masahiro Kanai Broad Institute, Cambridge, MA, United States
Mutaamba Maasha Broad Institute, Cambridge, MA, United States

Clinical Endpoint Development
Hannele Laivuori Institute for Molecular Medicine Finland, HiLIFE, University of Helsinki, Finland
Aki Havulinna Institute for Molecular Medicine Finland, HiLIFE, University of Helsinki, Finland
Susanna Lemmelä Institute for Molecular Medicine Finland, HiLIFE, University of Helsinki, Finland
Tuomo Kiiskinen Institute for Molecular Medicine Finland, HiLIFE, University of Helsinki, Finland
L. Elisa Lahtela Institute for Molecular Medicine Finland, HiLIFE, University of Helsinki, Finland
Matti Peura Institute for Molecular Medicine Finland, HiLIFE, University of Helsinki, Finland

Communication
Mari Kaunisto Institute for Molecular Medicine Finland, HiLIFE, University of Helsinki, Finland

Data Management and IT Infrastructure
Elina Kilpeläinen Institute for Molecular Medicine Finland, HiLIFE, University of Helsinki, Finland
Timo P. Sipilä Institute for Molecular Medicine Finland, HiLIFE, University of Helsinki, Finland
Georg Brein Institute for Molecular Medicine Finland, HiLIFE, University of Helsinki, Finland
Oluwaseun A. Dada Institute for Molecular Medicine Finland, HiLIFE, University of Helsinki, Finland
Awaisa Ghazal Institute for Molecular Medicine Finland, HiLIFE, University of Helsinki, Finland
Anastasia Shcherban Institute for Molecular Medicine Finland, HiLIFE, University of Helsinki, Finland

Genotyping
Kati Donner Institute for Molecular Medicine Finland, HiLIFE, University of Helsinki, Finland
Timo P. Sipilä Institute for Molecular Medicine Finland, HiLIFE, University of Helsinki, Finland

Sample Collection Coordination
Anu Loukola Helsinki Biobank / Helsinki University and Hospital District of Helsinki and Uusimaa, Helsinki

Sample Logistics

Päivi Laiho	THL Biobank / The National Institute of Health and Welfare Helsinki, Finland
Tuuli Sistonen	THL Biobank / The National Institute of Health and Welfare Helsinki, Finland
Essi Kaiharju	THL Biobank / The National Institute of Health and Welfare Helsinki, Finland
Markku Laukkanen	THL Biobank / The National Institute of Health and Welfare Helsinki, Finland
Elina Järvensivu	THL Biobank / The National Institute of Health and Welfare Helsinki, Finland
Sini Lähteenmäki	THL Biobank / The National Institute of Health and Welfare Helsinki, Finland
Lotta Männikkö	THL Biobank / The National Institute of Health and Welfare Helsinki, Finland
Regis Wong	THL Biobank / The National Institute of Health and Welfare Helsinki, Finland

Registry Data Operations

Hannele Mattsson	THL Biobank / The National Institute of Health and Welfare Helsinki, Finland
Kati Kristiansson	THL Biobank / The National Institute of Health and Welfare Helsinki, Finland
Susanna Lemmelä	Institute for Molecular Medicine Finland, HiLIFE, University of Helsinki, Finland
Sami Koskelainen	THL Biobank / The National Institute of Health and Welfare Helsinki, Finland
Tero Hiekkalinna	THL Biobank / The National Institute of Health and Welfare Helsinki, Finland
Teemu Paajanen	THL Biobank / The National Institute of Health and Welfare Helsinki, Finland

Sequencing Informatics

Priit Palta	Institute for Molecular Medicine Finland, HiLIFE, University of Helsinki, Finland
Kalle Pärn	Institute for Molecular Medicine Finland, HiLIFE, University of Helsinki, Finland
Shuang Luo	Institute for Molecular Medicine Finland, HiLIFE, University of Helsinki, Finland
Vishal Sinha	Institute for Molecular Medicine Finland, HiLIFE, University of Helsinki, Finland

Trajectory Team

Tarja Laitinen	Pirkanmaa Hospital District, Tampere, Finland
Harri Siirtola	University of Tampere, Tampere, Finland
Javier Gracia-Tabuenca	University of Tampere, Tampere, Finland
Mika Helminen	University of Tampere, Tampere, Finland
Tiina Luukkaala	University of Tampere, Tampere, Finland
Iida Vähätalo	University of Tampere, Tampere, Finland

Data protection officer

Tero Jyrhämä	Institute for Molecular Medicine Finland, HiLIFE, University of Helsinki, Finland

FinBB - Finnish biobank cooperative

Marco Hautalahti

Laura Mustaniemi

Mirkka Koivusalo

Sarah Smith

Tom Southerington
