# Supplementary material for: Comprehensive genome-wide association study of different forms of hernia identifies more than 80 associated loci
Source: Nat Commun. 2022 Jun 9;13:3200. doi: 10.1038/s41467-022-30921-4 (PMC9184475; doi:10.1038/s41467-022-30921-4)
Supplement: Supplementary file 3 — Description of Additional Supplementary Files [file 41467_2022_30921_MOESM3_ESM.pdf]

Description of Additional Supplementary Files

File Name: Supplementary Data 1
Description: provides further details on the UK Biobank and follow-up study groups, composition of the combined hernia groups, Pearson correlation coefficients for the 7 analyzed hernia groups, genomic control factors applied to the different SAIGE GWAS scans, hernia case codes in the UK Biobank.

File Name: Supplementary Data 2
Description: gives the results from conditional and joint analyses of SAIGE results with GCTA-COJO at P < 5 × 10-8 in all, males and females.

File Name: Supplementary Data 3
Description: contains results from the inguinal hernia meta-analysis in all, males and females; collapsed loci derived from GCTA-COJO results and the inguinal hernia meta-analysis at P < 5 × 10-8; heterogeneity analysis of the collapsed loci; MultiPhen results for 81 identified loci; regression results for locus on chromosome 7q33 and potential new loci.

File Name: Supplementary Data 4
Description: provides results from MAGMA gene prioritization and FUMA GTEx gene expression analyses based on complete SAIGE GWAS results.

File Name: Supplementary Data 5
Description: gives DEPICT results based on associated variants with P < 1 × 10-5: gene prioritization, genesets and tissue enrichment, and Gene Network results for DEPICT genes.

File Name: Supplementary Data 6
Description: contains GARFIELD enrichment analyses (Hotspots, Peaks, Footprints, Genic, TFBS, Histone Modifications, Chromatin States and FAIRE) for the 7 analyzed hernia groups.

File Name: Supplementary Data 7
Description: provides bivariate LD score regression between the five different forms of hernia and 245 traits, between the hernias and six psychiatric disorders, Mendelian randomization, heritability estimates for the five forms of hernia.

File Name: Supplementary Data 8
Description: has the PLINK clumping results for associated variants with P < 1 × 10-5 used as input for DEPICT.
