# Supplementary material for: Comprehensive genome-wide association study of different forms of hernia identifies more than 80 associated loci
Source: Nat Commun. 2022 Jun 9;13:3200. doi: 10.1038/s41467-022-30921-4 (PMC9184475; doi:10.1038/s41467-022-30921-4)
Supplement: Supplementary file 12 — Reporting Summary [file 41467_2022_30921_MOESM12_ESM.pdf]

nature research

Corresponding author(s): Frank Geller

Last updated by author(s): Apr 5, 2022

Reporting Summary

Nature Research wishes to improve the reproducibility of the work that we publish. This form provides structure for consistency and transparency in reporting. For further information on Nature Research policies, see Authors & Referees and the Editorial Policy Checklist.

Statistics

For all statistical analyses, confirm that the following items are present in the figure legend, table legend, main text, or Methods section.

n/a	Confirmed	
☐	☒	The exact sample size (*n*) for each experimental group/condition, given as a discrete number and unit of measurement
☐	☒	A statement on whether measurements were taken from distinct samples or whether the same sample was measured repeatedly
☐	☒	The statistical test(s) used AND whether they are one- or two-sided<br>*Only common tests should be described solely by name; describe more complex techniques in the Methods section.*
☐	☒	A description of all covariates tested
☐	☒	A description of any assumptions or corrections, such as tests of normality and adjustment for multiple comparisons
☐	☒	A full description of the statistical parameters including central tendency (e.g. means) or other basic estimates (e.g. regression coefficient) AND variation (e.g. standard deviation) or associated estimates of uncertainty (e.g. confidence intervals)
☐	☒	For null hypothesis testing, the test statistic (e.g. *F*, *t*, *r*) with confidence intervals, effect sizes, degrees of freedom and *P* value noted<br>*Give P values as exact values whenever suitable.*
☒	☐	For Bayesian analysis, information on the choice of priors and Markov chain Monte Carlo settings
☒	☐	For hierarchical and complex designs, identification of the appropriate level for tests and full reporting of outcomes
☐	☒	Estimates of effect sizes (e.g. Cohen's *d*, Pearson's *r*), indicating how they were calculated

Our web collection on statistics for biologists contains articles on many of the points above.

Software and code

Policy information about availability of computer code

Data collection

No additional data collection was done for this study, so that no software was used for data collection.

Data analysis

All GWAS analyses were done with SAIGE version 0.29.4 (Zhou, W. et al.: Efficiently controlling for case-control imbalance and sample relatedness in large-scale genetic association studies. Nat. Genet. 50, 1335–1341, 2018).
The conditional and joint analyses were done with the COJO option in GCTA version 1.26.0 (Yang, J., Lee, S. H., Goddard, M. E. & Visscher, P. M. GCTA: a tool for genome-wide complex trait analysis. Am. J. Hum. Genet. 88, 76–82, 2011).
The meta-analysis of the inguinal hernia studies was done with METAL version 2011.03.25 (Willer, C. J., Li, Y. & Abecasis, G. R. METAL: Fast and efficient meta-analysis of genomewide association scans. Bioinformatics (2010) doi:10.1093/bioinformatics/btq340).
Modelling multiple hernia phenotypes was performed with MultiPhen version 2.0.2 (O'Reilly, P. F. et al. MultiPhen: joint model of multiple phenotypes can increase discovery in GWAS. PLoS One 7, e34861–e34861, 2012).
Pathway and enrichment analyses were performed with MAGMA (de Leeuw, C. A., Mooij, J. M., Heskes, T. & Posthuma, D. MAGMA: generalized gene-set analysis of GWAS data. PLoS Comput. Biol. 11, e1004219–e1004219, 2015), FUMA version 1.3.6 (Watanabe, K., Taskesen, E., van Bochoven, A. & Posthuma, D. Functional mapping and annotation of genetic associations with FUMA. Nat. Commun. 8, 1826, 2017), the analyses were run via the web on June 23 and 24 2020, and the specifications and results are available in the public results section with IDs 424 to 430 (https://fuma.ctglab.nl/browse, author Frank Geller, titles UK Biobank followed by the respective hernia group), DEPICT version 1 rel 194 (Pers, T. H. et al. Biological interpretation of genome-wide association studies using predicted gene functions. Nat. Commun. 6, 5890, 2015), GeneNetwork version 2.0 (https://www.genenetwork.nl/, accession date 12/15/2020, Deelen, P. et al. Improving the diagnostic yield of exome- sequencing by predicting gene–phenotype associations using large-scale gene expression analysis. Nat. Commun. (2019) doi:10.1038/s41467-019-10649-4). and GARFIELD version 2 (Iotchkova, V. et al. GARFIELD classifies disease-relevant genomic features through integration of functional annotations with association signals. Nat. Genet. 51, 343–353, 2019).
Genetic correlation was calculated with LD score regression (Bulik-Sullivan, B. et al. An atlas of genetic correlations across human diseases and traits. Nat. Genet. (2015) doi:10.1038/ng.3406).
Mendelian randomization analyses were carried out with the R packages MendelianRandomization version 0.5.0 and MRPRESSO version 1.0.
Heritability was estimated with LD score regression (Bulik-Sullivan, B. et al. LD Score Regression Distinguishes Confounding from

Polygenicity in Genome-Wide Association Studies. bioRxiv (2014) doi:10.1101/002931), HESS version 0.54-beta (Shi, H., Kichaev, G. & Pasaniuc, B. Contrasting the Genetic Architecture of 30 Complex Traits from Summary Association Data. Am. J. Hum. Genet. 99, 139–153, 2016), LDAK version 5.1 (Speed, D., Holmes, J. & Balding, D. J. Evaluating and improving heritability models using summary statistics. Nat. Genet. 2020 52, 458–462), and transformed to the liability scale with GEAR (https://sourceforge.net/p/gbchen/wiki/Hong%20Lee's%20Transformation%20for%20Heritability/).
R packages and additional basic analyses were run in R version 3.2.0 or a specific version given with the code (R Core Team. R: A Language and Environment for Statistical Computing. https://www.R-project.org, 2019).

For manuscripts utilizing custom algorithms or software that are central to the research but not yet described in published literature, software must be made available to editors/reviewers. We strongly encourage code deposition in a community repository (e.g. GitHub). See the Nature Research guidelines for submitting code & software for further information.

Data

Policy information about availability of data

All manuscripts must include a data availability statement. This statement should provide the following information, where applicable:

- Accession codes, unique identifiers, or web links for publicly available datasets
- A list of figures that have associated raw data
- A description of any restrictions on data availability

The GWAS data from the UK Biobank analyses in this study have been deposited on the Danish National Biobank website (https://www.danishnationalbiobank.com/gwas). The remaining data generated in this study are provided in the Supplementary Information and Source Data file. Source data are provided with this paper.

Field-specific reporting

Please select the one below that is the best fit for your research. If you are not sure, read the appropriate sections before making your selection.

☒ Life sciences ☐ Behavioural & social sciences ☐ Ecological, evolutionary & environmental sciences

For a reference copy of the document with all sections, see nature.com/documents/nr-reporting-summary-flat.pdf

Life sciences study design

All studies must disclose on these points even when the disclosure is negative.

Sample size	*Sample sizes in the underlying studies were determined based on the phenotypic information available for genotyped individuals. The numbers are given in the method section. The numbers of cases and controls per study group are displayed in Figure 1.*
Data exclusions	*Individuals with hernia were generally excluded from the controls.*
Replication	*The findings from the GWAS of inguinal hernia were replicated in a follow-up study in four additional study groups.*
Randomization	*NA. The study is based on data from research projects and large Biobanks and not related to any clinical trials.*
Blinding	*NA. Participant data on disease were retrieved from health care records and interview data, there was no clinical trial involved.*

Behavioural & social sciences study design

All studies must disclose on these points even when the disclosure is negative.

Study description	*Briefly describe the study type including whether data are quantitative, qualitative, or mixed-methods (e.g. qualitative cross-sectional, quantitative experimental, mixed-methods case study).*
Research sample	*State the research sample (e.g. Harvard university undergraduates, villagers in rural India) and provide relevant demographic information (e.g. age, sex) and indicate whether the sample is representative. Provide a rationale for the study sample chosen. For studies involving existing datasets, please describe the dataset and source.*
Sampling strategy	*Describe the sampling procedure (e.g. random, snowball, stratified, convenience). Describe the statistical methods that were used to predetermine sample size OR if no sample-size calculation was performed, describe how sample sizes were chosen and provide a rationale for why these sample sizes are sufficient. For qualitative data, please indicate whether data saturation was considered, and what criteria were used to decide that no further sampling was needed.*
Data collection	*Provide details about the data collection procedure, including the instruments or devices used to record the data (e.g. pen and paper, computer, eye tracker, video or audio equipment) whether anyone was present besides the participant(s) and the researcher, and whether the researcher was blind to experimental condition and/or the study hypothesis during data collection.*
Timing	*Indicate the start and stop dates of data collection. If there is a gap between collection periods, state the dates for each sample cohort.*
Data exclusions	*If no data were excluded from the analyses, state so OR if data were excluded, provide the exact number of exclusions and the rationale*

Data exclusions	*behind them, indicating whether exclusion criteria were pre-established.*
Non-participation	*State how many participants dropped out/declined participation and the reason(s) given OR provide response rate OR state that no participants dropped out/declined participation.*
Randomization	*If participants were not allocated into experimental groups, state so OR describe how participants were allocated to groups, and if allocation was not random, describe how covariates were controlled.*

Ecological, evolutionary & environmental sciences study design

All studies must disclose on these points even when the disclosure is negative.

Study description	*Briefly describe the study. For quantitative data include treatment factors and interactions, design structure (e.g. factorial, nested, hierarchical), nature and number of experimental units and replicates.*
Research sample	*Describe the research sample (e.g. a group of tagged Passer domesticus, all Stenocereus thurberi within Organ Pipe Cactus National Monument), and provide a rationale for the sample choice. When relevant, describe the organism taxa, source, sex, age range and any manipulations. State what population the sample is meant to represent when applicable. For studies involving existing datasets, describe the data and its source.*
Sampling strategy	*Note the sampling procedure. Describe the statistical methods that were used to predetermine sample size OR if no sample-size calculation was performed, describe how sample sizes were chosen and provide a rationale for why these sample sizes are sufficient.*
Data collection	*Describe the data collection procedure, including who recorded the data and how.*
Timing and spatial scale	*Indicate the start and stop dates of data collection, noting the frequency and periodicity of sampling and providing a rationale for these choices. If there is a gap between collection periods, state the dates for each sample cohort. Specify the spatial scale from which the data are taken*
Data exclusions	*If no data were excluded from the analyses, state so OR if data were excluded, describe the exclusions and the rationale behind them, indicating whether exclusion criteria were pre-established.*
Reproducibility	*Describe the measures taken to verify the reproducibility of experimental findings. For each experiment, note whether any attempts to repeat the experiment failed OR state that all attempts to repeat the experiment were successful.*
Randomization	*Describe how samples/organisms/participants were allocated into groups. If allocation was not random, describe how covariates were controlled. If this is not relevant to your study, explain why.*
Blinding	*Describe the extent of blinding used during data acquisition and analysis. If blinding was not possible, describe why OR explain why blinding was not relevant to your study.*

Did the study involve field work? ☐ Yes ☐ No

Field work, collection and transport

Field conditions	*Describe the study conditions for field work, providing relevant parameters (e.g. temperature, rainfall).*
Location	*State the location of the sampling or experiment, providing relevant parameters (e.g. latitude and longitude, elevation, water depth).*
Access and import/export	*Describe the efforts you have made to access habitats and to collect and import/export your samples in a responsible manner and in compliance with local, national and international laws, noting any permits that were obtained (give the name of the issuing authority, the date of issue, and any identifying information).*
Disturbance	*Describe any disturbance caused by the study and how it was minimized.*

Reporting for specific materials, systems and methods

We require information from authors about some types of materials, experimental systems and methods used in many studies. Here, indicate whether each material, system or method listed is relevant to your study. If you are not sure if a list item applies to your research, read the appropriate section before selecting a response.

Materials & experimental systems

n/a	Involved in the study
☒	☐ Antibodies
☒	☐ Eukaryotic cell lines
☒	☐ Palaeontology
☒	☐ Animals and other organisms
☐	☒ Human research participants
☒	☐ Clinical data

Methods

n/a	Involved in the study
☒	☐ ChIP-seq
☒	☐ Flow cytometry
☒	☐ MRI-based neuroimaging

Antibodies

Antibodies used	*Describe all antibodies used in the study; as applicable, provide supplier name, catalog number, clone name, and lot number.*
Validation	*Describe the validation of each primary antibody for the species and application, noting any validation statements on the manufacturer's website, relevant citations, antibody profiles in online databases, or data provided in the manuscript.*

Eukaryotic cell lines

Policy information about cell lines

Cell line source(s)	*State the source of each cell line used.*
Authentication	*Describe the authentication procedures for each cell line used OR declare that none of the cell lines used were authenticated.*
Mycoplasma contamination	*Confirm that all cell lines tested negative for mycoplasma contamination OR describe the results of the testing for mycoplasma contamination OR declare that the cell lines were not tested for mycoplasma contamination.*
Commonly misidentified lines (See ICLAC register)	*Name any commonly misidentified cell lines used in the study and provide a rationale for their use.*

Palaeontology

Specimen provenance	*Provide provenance information for specimens and describe permits that were obtained for the work (including the name of the issuing authority, the date of issue, and any identifying information).*
Specimen deposition	*Indicate where the specimens have been deposited to permit free access by other researchers.*
Dating methods	*If new dates are provided, describe how they were obtained (e.g. collection, storage, sample pretreatment and measurement), where they were obtained (i.e. lab name), the calibration program and the protocol for quality assurance OR state that no new dates are provided.*

☐ Tick this box to confirm that the raw and calibrated dates are available in the paper or in Supplementary Information.

Animals and other organisms

Policy information about studies involving animals; ARRIVE guidelines recommended for reporting animal research

Laboratory animals	*For laboratory animals, report species, strain, sex and age OR state that the study did not involve laboratory animals.*
Wild animals	*Provide details on animals observed in or captured in the field; report species, sex and age where possible. Describe how animals were caught and transported and what happened to captive animals after the study (if killed, explain why and describe method; if released, say where and when) OR state that the study did not involve wild animals.*
Field-collected samples	*For laboratory work with field-collected samples, describe all relevant parameters such as housing, maintenance, temperature, photoperiod and end-of-experiment protocol OR state that the study did not involve samples collected from the field.*
Ethics oversight	*Identify the organization(s) that approved or provided guidance on the study protocol, OR state that no ethical approval or guidance was required and explain why not.*

Note that full information on the approval of the study protocol must also be provided in the manuscript.

Human research participants

Policy information about studies involving human research participants

Population characteristics	*The UK Biobank is a large-scale biomedical database and research resource containing genetic, lifestyle and health information*

Population characteristics

from half a million UK participants. Imputed genotypes and phenotype data are available for research. In brief, only genetic markers present on both the UK BiLEVE and UK Biobank Axiom arrays, passing QC in all batches and with a missingness < 5% and a minor allele frequency (MAF) > 0.0001 were considered, which resulted in a dataset with 670,739 autosomal markers in 487,442 samples. We used the provided imputed genotype data and restricted the analyses to well imputed variants (info > 0.8) with a MAF > 0.5% in 408,595 individuals of British ancestry. The questionnaire and hospital codes used for the five hernia types are listed in Supplementary Data 1. The combined groups of any hernia and any hernia excluding diaphragmatic hernia also included 4082 records of unspecific abdominal hernia repair from the interview on operations.
FinnGen (https://www.finngen.fi/en) is a large biobank study that aims to genotype 500,000 Finns and combine this data with longitudinal registry data including The National Hospital Discharge Registry, Causes of Death Registry and medication reimbursement registries, all these linked by unique national personal identification codes. FinnGen includes prospective and retrospective epidemiological and disease-based cohorts as well as hospital biobank samples. The methods are described in detail online (https://finngen.gitbook.io/documentation/methods/cohort-description). In short, individuals were genotyped with Illumina and Affymetrix chip arrays (Illumina Inc., San Diego, and Thermo Fisher Scientific, Santa Clara, CA, USA). In sample-wise quality control, individuals with ambiguous gender, high genotype missingness (> 5%), excess heterozygosity (+/- 4SD) and non-Finnish ancestry were excluded. In variant-wise quality control variants with high missingness (> 2%), deviation from Hardy Weinberg equilibrium ($P < 1 \times 10^{-6}$) and minor allele count < 3 were excluded. Genotype data were imputed using the population-specific SISu v3 imputation reference panel of 3,775 whole genomes. The fifth data freeze (spring 2020) used in this study consists of 218,792 individuals. Inguinal hernia cases were identified via The National Hospital Discharge Registry and Causes of Death Registry. Association analysis of the imputed SNPs was performed with SAIGE, adjusting for age, sex, the first 10 principal components and genotyping batch. The analysis had 17,096 cases and 190,557 controls.
Estonian Biobank samples were genotyped in Core Genotyping Lab of Institute of Genomics, University of Tartu using Illumina GSAv1.0, GSAv2.0, and GSAv2.0_EST arrays. Altogether 155,772 samples were genotyped and PLINK format files were created using Illumina GenomeStudio v2.0.4. Individuals were excluded from the analysis if their call-rate was < 95% or sex defined based on heterozygosity of X chromosome did not match sex in phenotype data. Variants were filtered by call-rate < 95% and HWE P value < 1e-4 (autosomal variants only). Variant positions were updated to build 37 and all variants were changed to be from TOP strand using tools and reference files provided in https://www.well.ox.ac.uk/~wrayner/strand/ webpage. Before imputation variants with MAF < 1% and indels were removed. QC was carried out using PLINK v1.9 and several in-house R and PERL scripts. After QC the dataset contained 154,201 samples for imputation. Prephasing was done using Eagle v2.3 and imputation was done using Beagle v.28Sep18.793 with effective population size ne=20,000. Estonian population specific imputation reference of 2,297 whole genome sequencing samples were used. Analyses were restricted to individuals with European ancestry. Inguinal hernia cases were identified from electronic health records (ICD-10 codes K40, K40.0, K40.1, K40.2, K40.3, K40.4 and K40.9). Association analysis of the imputed SNPs was performed with SAIGE, adjusting for year of birth, sex and the first six principal components. The analysis had 3,151 cases and 138,028 controls.
The iPSYCH study group included 78,050 individuals with GWAS data from the Illumina Infinium PsychChip v1.0. Data cleaning involved filtering out SNPs that had a MAF of < 0.01 or deviated from Hardy-Weinberg equilibrium ($P < 1 \times 10^{-6}$). Structural variants, tri-allelic SNPs, non-autosomal SNPs, and SNPs that were strand ambiguous (A/T or C/G) or did not uniquely align to the genome were also excluded, resulting in an imputation backbone of 246,369 autosomal SNPs. A total of 364 samples failing basic QC (discordant sex information, more than 1% missing genotypes, abnormal heterozygosity, duplicate sample discordance) were also excluded. Based on the good quality SNPs, all individuals were phased in a single batch using SHAPEIT3 and imputed in 10 batches using IMPUTE2 with reference haplotypes from the 1000 genomes project phase 3. Prior to analysis, we filtered out participants who were (1) of non-European ancestries, (2) related to another participant in the sample (corresponding to an IBD proportion >0.1875). We balanced the ratio of inguinal hernia cases to controls, so that it was the same within each of the 12 sex-specific groups of the iPSYCH study (5 psychiatric disorders: schizophrenia, affective disorder, ADHD, autism, and anorexia nervosa, as well as population controls). Inguinal hernia cases were identified from the National Patient Registry (ICD8 codes 550, 552, ICD-10 codes K40). Association analysis of imputed SNPs was performed with SAIGE, adjusting for year of birth, sex and the first 6 PCs. The analysis had 2,557 cases and 35,538 controls.
We used cases and controls from a Danish diverticular disease study. Samples were genotyped with the Illumina OmniExpress-24v1-2_A1 array and we applied the following quality control steps, with PLINK v1.90b3o, in order to sequentially remove (1) variant probes not uniquely aligned to the genome; (2) variants not present in dbSNP v.146, (3) variants with conflicting reference alleles between Illumina and dbSNP; (4) variants with missingness >5%, (5) variants that failed Hardy-Weinberg test at $P < 1\times 10^{-6}$, (6) variants with $p < 0.01$ in tests of differences in missingness between cases and controls; (7) variants with minor allele frequency < 1%, (8) samples with discordant sex between registry records and genotypes; and (9) samples with missingness > 2%. We also removed related individuals up until third degree relatives (kinship coefficient Phi > 0.05 estimated with KING as implemented in VCFtools version 0.1.12b). European ancestry outliers were removed based on principal component analysis of all our cases and controls combined with the European samples from the Human Genome Diversity Project using the R package SNPRelate v1.2.0. Outliers were defined based on the first two principal components, excluding samples where the distance between at least one principal component and its mean was larger than 6 times the interquartile range. This outlier removal procedure was repeated until no more samples fulfilled the criterion for removal. After these steps, we imputed unobserved genotypes with phased haplotypes from the Haplotype Reference Consortium panel version r1.1 on the imputation server at the Wellcome Trust Sanger Institute (https://imputation.sanger.ac.uk/). Inguinal hernia cases were defined as having an ICD-10 code K40 (and sub-codes) and/or ICD-8 codes 550 and/or 552 (and-sub-codes). Association analysis of imputed SNPs was performed with SAIGE, adjusting for year of birth, sex and diverticular disease status. The analysis had 999 inguinal hernia cases and 7768 controls.

Recruitment

The study groups are from general Biobanks (UK Biobank, FinnGen and Estonian Biobank) and studies with a specific interest in diseases (iPSYCH, diverticular disease). For the latter, the analyses were adjusted by using the same ratio of inguinal hernia cases : controls in each of the iPSYCH disease categories and the controls, and by including diverticular disease status as a covariate in the model. An additional sensitivity analysis for the diverticular disease studies is provided in the Supplementary Note.

Ethics oversight

Participants of the UK Biobank have given written informed consent and the UK Biobank has approval from the North West Multicentre Research Ethics Committee as a Research Tissue Bank (RTB), meaning that researchers do not require separate ethical clearance and can operate under the RTB approval (https://www.ukbiobank.ac.uk/learn-more-about-uk-biobank/about-us/ethics). This study was approved by the UK Biobank (project ID 33395).
Patients and control subjects in FinnGen provided informed consent for biobank research, based on the Finnish Biobank Act.

Alternatively, separate research cohorts, collected prior the Finnish Biobank Act came into effect (in September 2013) and start of FinnGen (August 2017), were collected based on study-specific consents and later transferred to the Finnish biobanks after approval by Fimea, the National Supervisory Authority for Welfare and Health. Recruitment protocols followed the biobank protocols approved by Fimea. The Coordinating Ethics Committee of the Hospital District of Helsinki and Uusimaa (HUS) approved the FinnGen study protocol Nr HUS/990/2017. The FinnGen study is approved by Finnish Institute for Health and Welfare (permit numbers: THL/2031/6.02.00/2017, THL/1101/5.05.00/2017, THL/341/6.02.00/2018, THL/2222/6.02.00/2018, THL/283/6.02.00/2019, THL/1721/5.05.00/2019, THL/1524/5.05.00/2020, and THL/2364/14.02/2020), Digital and population data service agency (permit numbers: VRK43431/2017-3, VRK/6909/2018-3, VRK/4415/2019-3), the Social Insurance Institution (permit numbers: KELA 58/522/2017, KELA 131/522/2018, KELA 70/522/2019, KELA 98/522/2019, KELA 138/522/2019, KELA 2/522/2020, KELA 16/522/2020 and Statistics Finland (permit numbers: TK-53-1041-17 and TK-53-90-20). The Biobank Access Decisions for FinnGen samples and data utilized in FinnGen Data Freeze 6 include: THL Biobank BB2017_55, BB2017_111, BB2018_19, BB_2018_34, BB_2018_67, BB2018_71, BB2019_7, BB2019_8, BB2019_26, BB2020_1, Finnish Red Cross Blood Service Biobank 7.12.2017, Helsinki Biobank HUS/359/2017, Auria Biobank AB17-5154, Biobank Borealis of Northern Finland_2017_1013, Biobank of Eastern Finland 1186/2018, Finnish Clinical Biobank Tampere MH0004, Central Finland Biobank 1-2017, and Terveystalo Biobank STB 2018001.
The use of the Estonian Biobank data in this study was approved by the Research Ethics Committee of the University of Tartu (Approval number 288/M-18).
The Danish Scientific Ethics Committee, the Danish Data Protection Agency and the Danish Neonatal Screening Biobank Steering Committee approved the iPSYCH study.
The Danish diverticular disease study was approved by the Scientific Ethics Committee of the Capital Region of Denmark (H-16016406) and the Danish Data Protection Agency. The Scientific Ethics Committee granted exemption from obtaining informed consent from participants as the study was based on the biobank material.

Note that full information on the approval of the study protocol must also be provided in the manuscript.

Clinical data

Policy information about clinical studies
All manuscripts should comply with the ICMJE guidelines for publication of clinical research and a completed CONSORT checklist must be included with all submissions.

Clinical trial registration	*Provide the trial registration number from ClinicalTrials.gov or an equivalent agency.*
Study protocol	*Note where the full trial protocol can be accessed OR if not available, explain why.*
Data collection	*Describe the settings and locales of data collection, noting the time periods of recruitment and data collection.*
Outcomes	*Describe how you pre-defined primary and secondary outcome measures and how you assessed these measures.*

ChIP-seq

Data deposition

☐ Confirm that both raw and final processed data have been deposited in a public database such as GEO.

☐ Confirm that you have deposited or provided access to graph files (e.g. BED files) for the called peaks.

Data access links<br>*May remain private before publication.*	*For "Initial submission" or "Revised version" documents, provide reviewer access links. For your "Final submission" document, provide a link to the deposited data.*
Files in database submission	*Provide a list of all files available in the database submission.*
Genome browser session<br>(e.g. UCSC)	*Provide a link to an anonymized genome browser session for "Initial submission" and "Revised version" documents only, to enable peer review. Write "no longer applicable" for "Final submission" documents.*

Methodology

Replicates	*Describe the experimental replicates, specifying number, type and replicate agreement.*
Sequencing depth	*Describe the sequencing depth for each experiment, providing the total number of reads, uniquely mapped reads, length of reads and whether they were paired- or single-end.*
Antibodies	*Describe the antibodies used for the ChIP-seq experiments; as applicable, provide supplier name, catalog number, clone name, and lot number.*
Peak calling parameters	*Specify the command line program and parameters used for read mapping and peak calling, including the ChIP, control and index files used.*
Data quality	*Describe the methods used to ensure data quality in full detail, including how many peaks are at FDR 5% and above 5-fold enrichment.*
Software	*Describe the software used to collect and analyze the ChIP-seq data. For custom code that has been deposited into a community repository, provide accession details.*

Flow Cytometry

Plots

Confirm that:

- [ ] The axis labels state the marker and fluorochrome used (e.g. CD4-FITC).
- [ ] The axis scales are clearly visible. Include numbers along axes only for bottom left plot of group (a 'group' is an analysis of identical markers).
- [ ] All plots are contour plots with outliers or pseudocolor plots.
- [ ] A numerical value for number of cells or percentage (with statistics) is provided.

Methodology

Sample preparation	*Describe the sample preparation, detailing the biological source of the cells and any tissue processing steps used.*
Instrument	*Identify the instrument used for data collection, specifying make and model number.*
Software	*Describe the software used to collect and analyze the flow cytometry data. For custom code that has been deposited into a community repository, provide accession details.*
Cell population abundance	*Describe the abundance of the relevant cell populations within post-sort fractions, providing details on the purity of the samples and how it was determined.*
Gating strategy	*Describe the gating strategy used for all relevant experiments, specifying the preliminary FSC/SSC gates of the starting cell population, indicating where boundaries between "positive" and "negative" staining cell populations are defined.*

- [ ] Tick this box to confirm that a figure exemplifying the gating strategy is provided in the Supplementary Information.

Magnetic resonance imaging

Experimental design

Design type	*Indicate task or resting state; event-related or block design.*
Design specifications	*Specify the number of blocks, trials or experimental units per session and/or subject, and specify the length of each trial or block (if trials are blocked) and interval between trials.*
Behavioral performance measures	*State number and/or type of variables recorded (e.g. correct button press, response time) and what statistics were used to establish that the subjects were performing the task as expected (e.g. mean, range, and/or standard deviation across subjects).*

Acquisition

Imaging type(s)	*Specify: functional, structural, diffusion, perfusion.*
Field strength	*Specify in Tesla*
Sequence & imaging parameters	*Specify the pulse sequence type (gradient echo, spin echo, etc.), imaging type (EPI, spiral, etc.), field of view, matrix size, slice thickness, orientation and TE/TR/flip angle.*
Area of acquisition	*State whether a whole brain scan was used OR define the area of acquisition, describing how the region was determined.*

Diffusion MRI ☐ Used ☐ Not used

Preprocessing

Preprocessing software	*Provide detail on software version and revision number and on specific parameters (model/functions, brain extraction, segmentation, smoothing kernel size, etc.).*
Normalization	*If data were normalized/standardized, describe the approach(es): specify linear or non-linear and define image types used for transformation OR indicate that data were not normalized and explain rationale for lack of normalization.*
Normalization template	*Describe the template used for normalization/transformation, specifying subject space or group standardized space (e.g. original Talairach, MNI305, ICBM152) OR indicate that the data were not normalized.*
Noise and artifact removal	*Describe your procedure(s) for artifact and structured noise removal, specifying motion parameters, tissue signals and physiological signals (heart rate, respiration).*
Volume censoring	*Define your software and/or method and criteria for volume censoring, and state the extent of such censoring.*

Statistical modeling & inference

Model type and settings

Specify type (mass univariate, multivariate, RSA, predictive, etc.) and describe essential details of the model at the first and second levels (e.g. fixed, random or mixed effects; drift or auto-correlation).

Effect(s) tested

Define precise effect in terms of the task or stimulus conditions instead of psychological concepts and indicate whether ANOVA or factorial designs were used.

Specify type of analysis: ☐ Whole brain ☐ ROI-based ☐ Both

Statistic type for inference
(See Eklund et al. 2016)

Specify voxel-wise or cluster-wise and report all relevant parameters for cluster-wise methods.

Correction

Describe the type of correction and how it is obtained for multiple comparisons (e.g. FWE, FDR, permutation or Monte Carlo).

Models & analysis

n/a	Involved in the study
☐	☐ Functional and/or effective connectivity
☐	☐ Graph analysis
☐	☐ Multivariate modeling or predictive analysis

Functional and/or effective connectivity

Report the measures of dependence used and the model details (e.g. Pearson correlation, partial correlation, mutual information).

Graph analysis

Report the dependent variable and connectivity measure, specifying weighted graph or binarized graph, subject- or group-level, and the global and/or node summaries used (e.g. clustering coefficient, efficiency, etc.).

Multivariate modeling and predictive analysis

Specify independent variables, features extraction and dimension reduction, model, training and evaluation metrics.
